# Synthesis and Antiproliferative Activity of Thiazolyl-bis-pyrrolo[2,3-*b*]pyridines and Indolyl-thiazolyl-pyrrolo[2,3-*c*]pyridines, Nortopsentin Analogues

**DOI:** 10.3390/md13010460

**Published:** 2015-01-16

**Authors:** Anna Carbone, Barbara Parrino, Gloria Di Vita, Alessandro Attanzio, Virginia Spanò, Alessandra Montalbano, Paola Barraja, Luisa Tesoriere, Maria Antonia Livrea, Patrizia Diana, Girolamo Cirrincione

**Affiliations:** Biological, Chemical and Pharmaceutical Sciences and Technologies Department, STEBICEF, Università degli Studi di Palermo, via Archirafi 32, 90123 Palermo, Italy; E-Mails: anna.carbone@unipa.it (A.C.); barbara.parrino@unipa.it (B.P.); gloria.divita@unipa.it (G.D.V.); alessandro.attanzio@unipa.it (A.A.); virginia.spano@unipa.it (V.S.); alessandra.montalbano@unipa.it (A.M.); paola.barraja@unipa.it (P.B.); luisa.tesoriere@unipa.it (L.T.); maria.livrea@unipa.it (M.A.L.); patrizia.diana@unipa.it (P.D.)

**Keywords:** marine alkaloids, bis-indolyl alkaloids, nortopsentins, thiazolyl-bis-pyrrolo[2,3-*b*]pyridines, indolyl-thiazolyl-pyrrolo[2,3-*c*]pyridines, apoptosis, autophagic death, antiproliferative activity

## Abstract

Two new series of nortopsentin analogues, in which the imidazole ring of the natural product was replaced by thiazole and indole units were both substituted by 7-azaindole moieties or one indole unit was replaced by a 6-azaindole portion, were efficiently synthesized. Compounds belonging to both series inhibited the growth of HCT-116 colorectal cancer cells at low micromolar concentrations, whereas they did not affect the viability of normal-like intestinal cells. A compound of the former series induced apoptosis, evident as externalization of plasma membrane phosphatidylserine (PS), and changes of mitochondrial trans-membrane potential, while blocking the cell cycle in G2/M phase. In contrast, a derivative of the latter series elicited distinct responses in accordance with the dose. Thus, low concentrations (GI_30_) induced morphological changes characteristic of autophagic death with massive formation of cytoplasmic acid vacuoles without apparent loss of nuclear material, and with arrest of cell cycle at the G1 phase, whereas higher concentrations (GI_70_) induced apoptosis with arrest of cell cycle at the G1 phase.

## 1. Introduction

In recent years, a great deal of attention has been paid to the identification and development of new molecules based on natural or synthetic heterocyclic scaffolds as possible novel cancer therapeutic agents [1,2,3,4,5,6,7,8,9,10,11,12]. In particular, bis-indolyl alkaloids, characterized by two indole units bound to a spacer through their position 3, constitute a class of deep-sea sponge metabolites with potent biological activities such as antiinflammatory, antimicrobial, antiviral, and antitumor [13,14,15,16].

**Chart 1:**
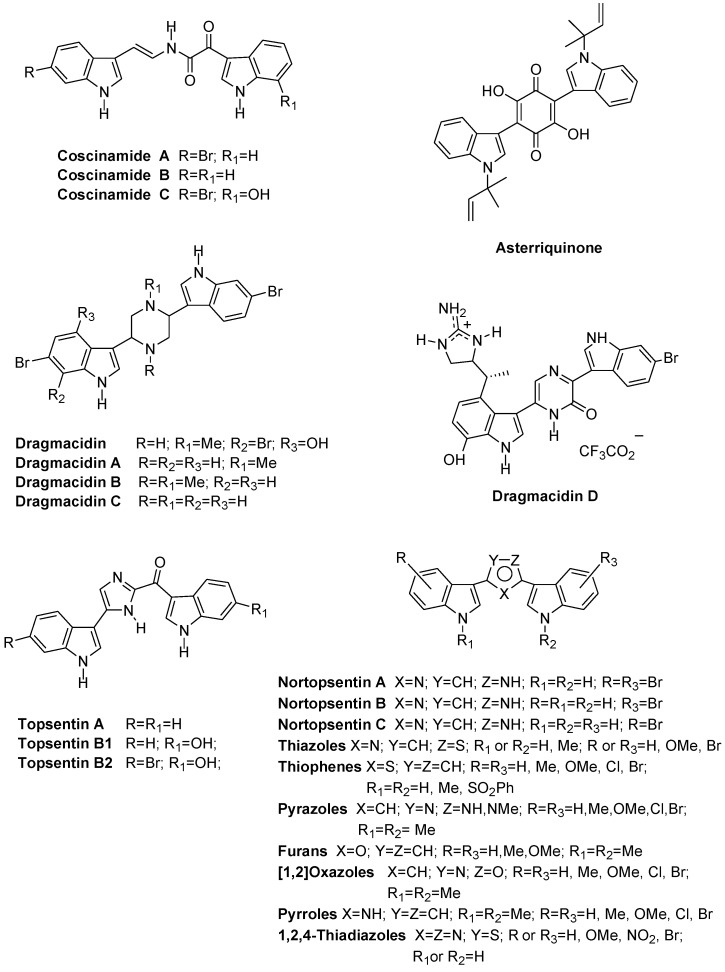
Bis-indolyl alkaloids and analogues.

The spacer structural profile can vary from a linear chain to carbocycles or heterocycles differently sized. Thus, coscinamides A–C, isolated from deep marine sponge *Coscinoderma* sp. bearing a linear chain as a spacer, showed HIV inhibitory activity (Chart 1) [17]. Asterriquinone, isolated from *Aspergillus fungi*, bears a quinone symmetrical structure and showed* in vivo* activity against Ehrlich carcinoma, ascites hepatoma AH13, and mouse P388 leukemia [18]. Dragmacidins have been isolated from a large number of deep water sponges such as *Dragmacidon*, *Halicortex*, *Spongosorites*, *Hexadella* and the tunicate *Didemnum candidum*, and present different spacers and diverse related activities. Dragmacidin and dragmacidins A–C, which exhibit the saturated six-membered heterocyclic link piperazine, showed modest cytotoxic activity [19,20,21]. Instead, a more complex member of this family, dragmacidin D, bearing a pyrazinone moiety as central core, exhibited several biological properties such as inhibition of serine-threonine protein phosphatases, antiviral, antimicrobial, and anticancer activities [22,23].

Topsentins A, B1 and B2, isolated from Mediterranean sponge *Topsentia genitrix*, bearing the five-membered ring spacer imidazole, showed antitumor and antiviral activities [24,25].

Nortopsentins A–C also bearing the imidazole ring as a spacer, were isolated from *Spongosorites ruetzleri*, and showed* in vitro* cytotoxicity against P388 cells (GI_50_, 4.5–20.7 μM). Replacement of the indole nitrogen with a methyl group led to derivatives that showed a significant improvement in cytotoxicity against P388 cells (GI_50_, 0.8–2.1 μM) [26,27].

Due to a great limitation in the use of the reservoir of marine organism that allow the isolation of very small amount of the biologically active substances from the natural material, several total synthesis of nortopsentins were proposed [28,29,30,31].

Moreover, due to the considerable activities shown, indolyl alkaloids have become an attractive field in medicinal chemistry and several dragmacidin analogues bearing six membered rings such as pyridine, pyrazine, pyrazinone and pyrimidine as spacer between the two indole units were synthesized. These analogues showed good antiproliferative activity against a wide range of human tumor cell lines [32,33,34,35]. Many papers reported the synthesis and the evaluation of the antiproliferative activity of nortopsentin analogues bearing five membered heterocycles which replaced the imidazole ring of the natural product such as bis-indolyl-thiophenes [36], -pyrazoles [37], -furans [38], -isoxazoles [38], -pyrroles [39], and -1,2,4-thiadiazoles [40]. Most of these analogues exhibited good antiproliferative activity against wide range of human tumor cell lines often reaching GI_50_ values at submicromolar level.

Moreover, the structural manipulation of the natural nortopsentins, beside the heterocyclic spacer, was extended to one or both indole units and led to 3-[(2-indolyl)-5-phenyl]pyridines and phenylthiazolyl-7-azaindole derivatives. Both these series of compounds showed antiproliferative activity against a wide range of human tumor cell lines in the micromolar-submicromolar range and were able to inhibit the activity of the cyclin-dependent kinase 1 (CDK1) with GI_50_ values lower than 1 μM [41,42].

**Chart 2:**
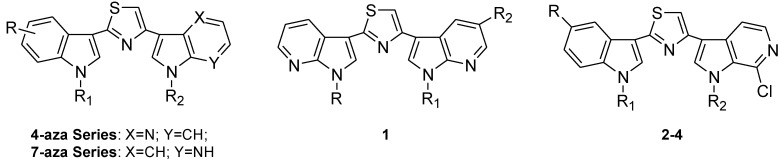
Nortopsentin aza-analogues.

More recently, due to the good results obtained by the aza-substitution of the indole moiety, 3-[2-(1*H*-indol-3-yl)-1,3-thiazol-4-yl]-1*H*-4-azaindole derivatives, in which the 4-azaindole ring substituted one indole system and the thiazole moiety replaced the imidazole nucleus of nortopsentin were synthesized and tested against a panel of cell lines with different histologic origin, including breast cancer, androgen-independent prostate cancer, pancreatic carcinoma and peritoneal mesothelioma (Chart 2). Four compounds showed GI_50_ values ranging from 2.20 to 19.36 μM, and were also able to inhibit CDK1 with GI_50_ in the range 0.64–0.87 μM. Moreover, the most active compound also reduced the cyclin B1-associated CDK1 kinase activity in a peritoneal mesothelioma cell line and increased by 4-fold and 3-fold caspase-9 and caspase-3 activity respectively [43].

Contemporaneously, 3-[2-(1*H*-indol-3-yl)-1,3-thiazol-4-yl)-1*H*-7-azaindoles, in which the spacer is constituted by the thiazole ring and one of the indole units is replaced by a 7-azaindole moiety were synthesized.

All compounds of this series were tested against the NCI full panel of human cancer cell lines and STO and MesoII cells, derived from human diffuse malignant peritoneal mesothelioma (DMPM). The most active compounds, that also act as CDK1 inhibitors, consistently reduced DMPM cell proliferation and induced a caspase-dependent apoptotic response, with a concomitant reduction of the expression of the active Thr^34^-phosphorylated form of the anti-apoptotic protein survivin. Moreover, the combined treatment of DMPM cells with the most active derivative and paclitaxel produced a synergistic cytotoxic effect, which was parallel by an enhanced apoptotic response. In the mouse model, i.p. administration of active derivatives was effective, resulting in a significant tumor volume inhibition of DMPM xenografts (range, 58%–75%) at well-tolerated doses, and two complete responses were observed in each treatment group [44].

In this paper, continuing our studies on indole and related azaindole systems [45,46,47,48,49], we report the synthesis of derivatives of type 1, nortopsentin analogues in which both indole units are replaced by a 7-azaindole moiety, and of derivatives of type 2–4 in which one indole unit was replaced by a 6-azaindole moiety. We also report the antiproliferative activity of these new nortopsentin analogues and studies directed to elucidate their mode of action.

## 2. Results and Discussion

### 2.1. Chemistry

**Scheme 1:**
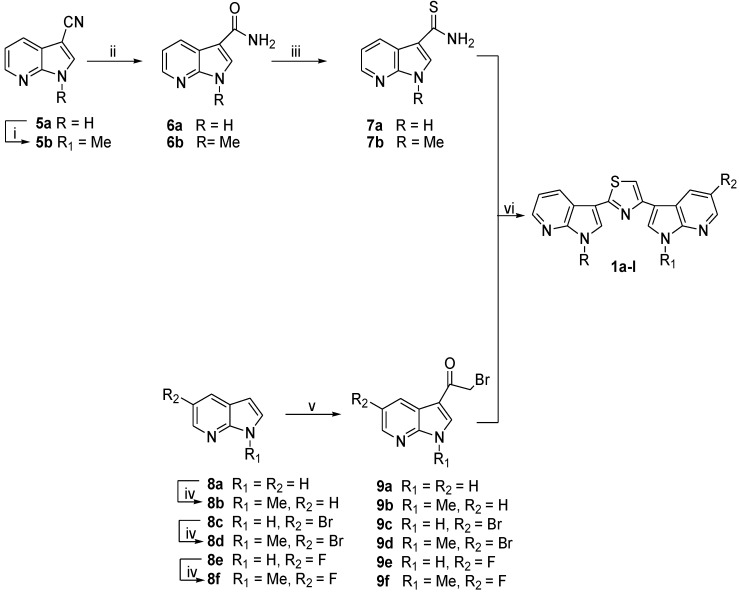
Synthesis of thiazolyl-bis-pyrrolo[2,3-*b*]pyridines **1a–l**.

**Scheme 2:**
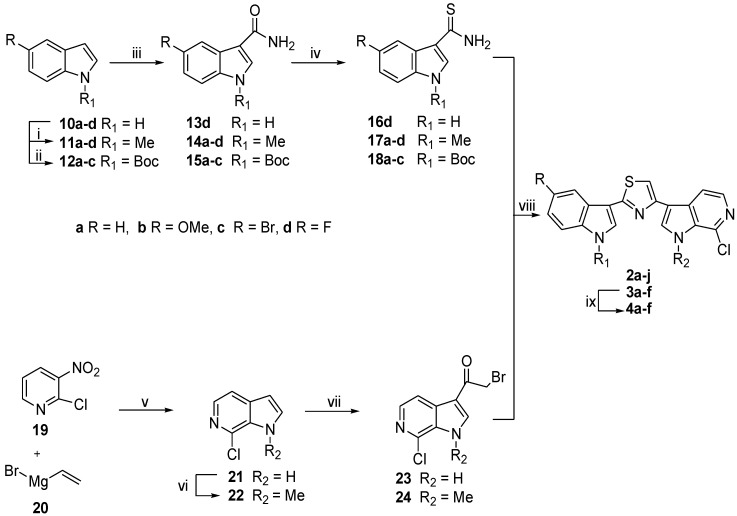
Synthesis of indolyl-thiazolyl-pyrrolo[2,3-*c*]pyridines** 2a–j**, **3a–f**, **4a–f**.

The synthesis of thiazolyl-bis-pyrrolo[2,3-*b*]pyridines of type **1** involved the construction of the thiazole ring by Hantzsch reaction between the two key intermediates pyrrolo[2,3-*b*]pyridine-carbothioamides **7** and 3-bromoacetyl**-**pyrrolo[2,3-*b*]pyridines **9** (Scheme 1). The Hantzsch reaction was also used for the synthesis of indolyl-thiazolyl-pyrrolo[2,3-*c*]pyridines of type **2**–**4**, but, in this case the key intermediates were indolocarbothioamides **16**–**18** and 3-bromoacetyl-pyrrolo[2,3-*c*]pyridines **23**, **24** (Scheme 2).

The starting material for the preparation of pyrrolo[2,3-*b*]pyridine-carbothioamides **7a**,**b** (Scheme 1) were the corresponding carboxamides **6a**,**b** conveniently prepared in excellent yields (95%–99%) by reaction of the corresponding pyrrolo[2,3-*b*]pyridine-3-carbonitrile **5a**,**b** with sulfuric acid and subsequent alkalinization. Treatment of carboxamides **6a**,**b** with Lawesson’s reagent in refluxing tetrahydrofuran (THF) gave the desired pyrrolo[2,3-*b*]pyridine-carbothioamides **7a**,**b** (88%–99%).

Pyrrolo[2,3-*b*]pyridines **8a**,**c**,**e** were converted into the corresponding *N*-methyl derivatives **8b**,**d**,**f** (60%–96%) using potassium *tert*-butoxide (*t*-BuOK), tris[2-(2-methoxyethoxy)ethyl]amine (TDA-1) as a catalyst and methyl iodide (MeI) in toluene [42].

3-Bromoacetyl-pyrrolo[2,3-*b*]pyridines **9a–f** were efficiently synthesized (70%–92%) by acylation of the corresponding pyrrolo-pyridines **8a–f** with bromoacetyl bromide (BrCOCH_2_Br) in the presence of aluminium chloride (AlCl_3_) in dichloromethane (DCM) (Scheme 1) [42]. Reaction of pyrrolo[2,3-*b*]pyridine-carbothioamides **7a**,**b** and 3-bromoacetyl-pyrrolo[2,3-*b*]pyridines **9a–f** in ethanol (EtOH) under reflux gave the desired thiazolyl-bis-pyrrolo[2,3-*b*]pyridines **1a–l** in good to excellent yields (60%–94%) (Scheme 1).

Reagents: (i) (a) *t*-BuOK, toluene, TDA-1, rt, 6 h; (b) MeI, rt, 1 h, 85%; (ii) (a) H_2_SO_4_, rt, 15–60 min; (b) NaOH, 95%–99%; (iii) Lawesson’s reagent, THF, reflux, 30 min, 88%–99%; (iv) (a) *t*-BuOK, toluene, TDA-1, rt, 3 h; (b) MeI, rt, 1 h, 60%–96%; (v) AlCl_3_, DCM, BrCOCH_2_Br, reflux, 40 min, 70%–92%; (vi) EtOH, reflux, 30 min, 60%–94%.

Indole-3-carbothioamides **16d**, **17a–d** and **18a–c** (Scheme 2) were obtained from the corresponding indoles **10d**, **11a–d**, and **12a–c** through the formation of amides **13d**, **14a–d** and **15a–c** as previously reported [43,44].

7-Chloro-1*H*-pyrrolo[2,3-*c*]pyridine **21** was obtained by reaction of 2-chloro-3-nitropyridine **19** with vinylmagnesium bromide **20** in THF under nitrogen atmosphere as previously reported [50].

Compound **21** was converted into the corresponding *N*-methylated derivative **22**, and both pyrrolo[2,3-*c*]pyridines were transformed into the corresponding 3-bromoacetyl-pyrrolo[2,3-*c*]pyridines **23**, **24** in excellent yields (88%–90%) by usual acylating procedure [42].

The reaction of indolocarbothioamides **16d**, **17a–d** and **18a–c** with 3-bromoacetyl-pyrrolo[2,3-*c*]pyridines **23**, **24** provided the indolyl-thiazolyl-pyrrolo[2,3-*c*]pyridines **2a–j** and **3a–f** in good to excellent yields (65%–98%) (Scheme 2). The subsequent deprotection of *N*-*tert*-butylcarboxylate derivatives **3a–f** using trifluoacetic acid (TFA) in DCM under reflux afforded, after neutralization with hydrogen carbonate (NaHCO_3_), the corresponding thiazoles **4a–f** in good to excellent yields (62%–99%) (Table 1).

**Table 1:** Substituted thiazolyl-bis-pyrrolo[2,3-*b*]pyridines **1a–l** and indolyl-thiazolyl-pyrrolo[2,3-*c*]pyridines **2a–j**, **3a–f**, **4a–f**.

Compd	R	R_1_	R_2_	Yield%	Compd	R	R_1_	R_2_	Yield%
**1a**	H	H	H	63	**2f**	OMe	Me	Me	79
**1b**	H	H	Br	60	**2g**	Br	Me	Me	98
**1c**	H	H	F	75	**2h**	F	Me	Me	95
**1d**	Me	H	H	90	**2i**	F	H	Me	82
**1e**	Me	H	Br	94	**2j**	F	H	H	66
**1f**	Me	H	F	90	**3a**	H	Boc	H	84
**1g**	Me	Me	H	60	**3b**	OMe	Boc	H	77
**1h**	Me	Me	Br	90	**3c**	Br	Boc	H	77
**1i**	Me	Me	F	60	**3d**	H	Boc	Me	91
**1j**	H	Me	H	85	**3e**	OMe	Boc	Me	92
**1k**	H	Me	Br	80	**3f**	Br	Boc	Me	91
**1l**	H	Me	F	72	**4a**	H	H	H	93
**2a**	H	Me	H	98	**4b**	OMe	H	H	62
**2b**	OMe	Me	H	65	**4c**	Br	H	H	98
**2c**	Br	Me	H	93	**4d**	H	H	Me	75
**2d**	F	Me	H	97	**4e**	OMe	H	Me	73
**2e**	H	Me	Me	96	**4f**	Br	H	Me	99

Reagents: (i) (a) *t*-BuOK, toluene, TDA-1, rt, 1–24 h; (b) MeI, rt, 0.5–1 h, 96%–98%; (ii) (Boc)_2_O, TEA, THF, reflux, 24–48 h, 90%–100%; (iii) (a) CSI, MeCN, 0 °C-rt, then rt 0.5–2 h or reflux 15 min; (b) 10% KOH, aq. acetone, 40%–60%; (iv) Lawesson’s reagent, toluene or benzene, reflux, 0.5–24 h, 90%–98%; (v) THF, −78 °C, 8 h, 33%; (vi) (a) *t*-BuOK, toluene, TDA-1, rt, 5 h; (b) MeI, rt, 2 h, 70%; (vii) AlCl_3_, DCM, BrCOCH_2_Br, reflux, 40 min, 88%–90%; (viii) EtOH, reflux, 1–3 h, 65%–98%; (ix) (a) TFA, DCM, reflux, 24 h; (b) aqueous NaHCO_3_, 62%–99%.

### 2.2. Biology

All the synthesized thiazoles **1a–l**, **2a–j**, **3a–f** and **4a–f** were evaluated by the National Cancer Institute (Bethesda MD) for cytotoxicity against the NCI-60 cell line panel using standard protocols [51]. Initially, all derivatives were tested at a single dose (10^−5^ M) on the full panel of approximately 60 human tumor cell lines derived from 9 human cancer cell types, that have been grouped in disease sub-panels including leukemia, non-small-cell lung, colon, central nervous system (CNS), melanoma, ovarian, renal, prostate and breast cancers (data not shown). Compounds **1k** and **4c** were further selected for full evaluation at five concentration levels (10^−4^–10^−8^ M).

The antitumor activity of compounds **1k** and **4c** was given by three parameters for each cell line: GI_50_ (GI_50_ is the molar concentration of the compound that inhibits 50% net cell growth), TGI (TGI is the molar concentration of the compound leading to total inhibition of net cell growth), and LC_50_ (LC_50_ is the molar concentration of the compound that induces 50% net cell death). The average values of mean graph midpoint (MG_MID) were calculated for each of these parameters. An evaluation of the data reported in Table 2 pointed out that compounds **1k** and **4c** exhibited antiproliferative activity against most of the human cell lines at GI_50_ values from micromolar to submicromolar (0.81–27.7 and 0.93–4.70 µM respectively).

**Table 2:** *In vitro* inhibition of cancer cell line growth by compounds **1k**, **4c**^ a^.

Cell Lines	GI_50_ (µM)		Cell Lines	GI_50_ (µM)		Cell Lines	GI_50_ (µM)	
	1k	4c		1k	4c		1k	4c
Leukemia			CNS Cancer			Renal Cancer		
CCRF-CEM	6.81	3.16	SF-268	6.01	4.06	786-0	9.65	1.37
HL-60(TB)	>100	2.64	SF-295	3.01	2.48	A498	10.9	1.56
K-562	8.76	2.73	SF-539	27.7	1.87	ACHN	2.35	2.02
MOLT-4	>100	3.02	SNB-19	6.74	3.25	CAKI-1	1.56	1.96
RPMI-8226	>100	4.03	SNB-75	2.18	2.37	RXF393	2.05	1.48
SR	ND ^b^	1.27	U251	2.70	2.05	SN12C	ND	3.35
						TK-10	3.77	4.16
**Non-Small Cell Lung Cancer**			**Melanoma**			UO-31	ND	0.93
A549/ATCC	2.59	3.83	LOX IMVI	4.26	1.63			
EKVK	1.27	3.11	MALME-3M	3.01	ND	**Prostate Cancer**		
HOP-62	2.39	2.11	M14	4.06	2.22	PC-3	4.35	3.86
HOP-92	5.03	2.43	MDA-MB-435	3.17	3.43	DU-145	3.51	1.76
NCI-H226	1.97	2.40	SK-MEL-2	19.8	4.09			
NCI-H23	2.80	2.42	SK-MEL-28	ND	1.85	**Breast Cancer**		
NCI-H322M	>100	3.54	SK-MEL-5	2.05	2.61	MCF7	6.77	2.20
NCI-H460	2.98	2.16	UACC-257	ND	2.68	MDA-MB-231/ATCC	3.02	1.68
NCI-H522	4.86	2.28	UACC-62	3.47	2.19	HS 578T	2.43	3.70
						BT-549	20.5	4.70
						T-47D	1.80	3.12
**Colon Cancer**			**Ovarian Cancer**			MDA-MB-468	0.81	1.18
COLO-205	ND	1.80	IGROV1	2.21	2.51			
HCC-2998	>100	2.22	OVCAR-3	2.91	3.45			
HCT-116	2.91	2.35	OVCAR-4	2.03	3.25			
HCT-15	13.7	1.40	OVCAR-5	>100	3.14			
HT29	6.75	2.66	OVCAR-8	3.72	3.65			
KM12	5.70	2.15	NCI/ADR-RES	3.88	2.71			
SW-620	4.46	1.92	SK-OV-3	3.25	2.78			

^a^ Data obtained from NCI’s* in vitro* disease-oriented tumor cells screen; ^b^ ND = not determined.

The indolyl-thiazolyl-pyrrolo[2,3-*c*]pyridine derivative **4c** resulted more active than thiazolyl-bis-pyrrolo[2,3-*b*]pyridines derivative **1k** in terms either of GI_50_ (mean value 2.59 and 5.26 µM respectively) and percentage of sensitive cell lines out of the total number of cell lines investigated (100% and 89%, respectively).

Moreover for compound **4c** positive TGI and LC_50_ values were observed with respect to a good number of cell lines (98% and 81%, respectively; data not shown).

Derivative **4c** was shown to be selective with respect to the renal cancer subpanel having all the subpanel cell lines GI_50_ in the range 0.93–4.16 µM. The most sensitive cell lines are UO-31 (GI_50_ 0.93 µM), 786-0 (GI_50_ 1.37 µM), RXF393 (GI_50_ 1.48 µM), and A498 (GI_50_ 1.56 µM). Cell lines sensitive to derivative **4c** were also SR (GI_50_ 1.27 µM) of leukemia, HCT-15 (GI_50_ 1.40 µM) of colon cancer, and MDA-MB-468 (GI_50_ 1.18 µM) of breast cancer subpanel.

Derivative **1k** showed selectivity with respect to MDA-MB-468 (GI_50_ 0.81 µM) of breast cancer, EKVK (GI_50_ 1.27 µM) of non small cell lung, and CAKI-1 (GI_50_ 1.56 µM) of renal cancer subpanel.

**Figure 1:**
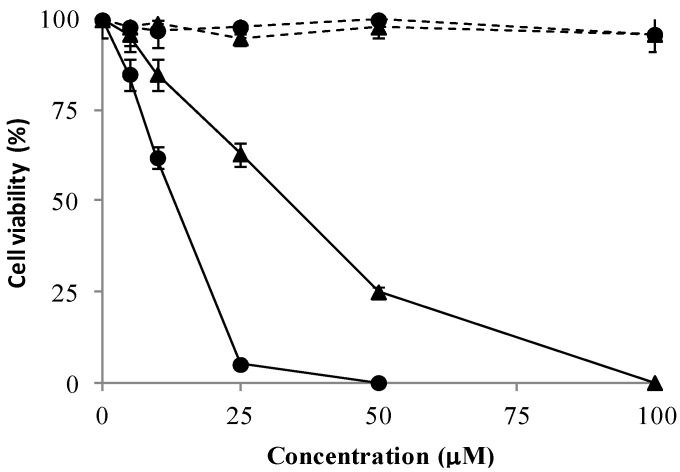
Effect of **1k** (triangle) and **4c** (circle) on the viability of human intestinal cell lines either tumoral (HCT-116; full line) or normal-like (differentiated Caco-2; dashed line). Cells were treated with the compounds **1k** and **4c** and cell survival was measured after 24 h by MTT assay in comparison to cells treated with vehicle alone (control). Values are the mean of three separate experiments in triplicate.

Selective toxicity of **1k **and **4c** nortopsentin analogues towards tumor cells was investigated. To this aim human HTC-116 colorectal carcinoma cells, against which both compounds exhibited a comparable antiproliferative effects (GI_50_ 2.91 and 2.35 μM, Table 2), and intestinal normal-like differentiated Caco-2 cells were exposed to the compounds for 24 h and viability compared. While both derivatives, in the range 5 to 100 μM, dose-dependently inhibited the intestinal HCT-116 cell proliferation, they did not affect the differentiated Caco-2 cell viability, suggesting tumor cells as the main target of their cytocidal action (Figure 1). GI_50_ values of **1k** or **4c** calculated after 24 h treatment of HCT-116 cells, were 33.55 ± 2.31 μM and 13.15 ± 0.95 μM, respectively. Taking in account the GI_50_ values measured by NCI on the same cell line after 72 h treatment, the cytotoxic effects of the nortopsentin analogues appeared time-dependent.

#### 2.2.1. Cell Cycle Alterations

**Figure 2:**
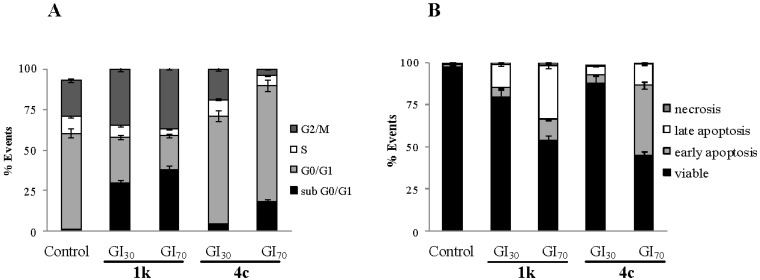
Effect of **1k** and **4c** on the cell cycle distribution and apoptosis of HCT-116 cells. Flow cytometric analysis of propidium iodide-stained cells (**A**) and percentage of Annexin V/propidium iodide (PI) double-stained cells (**B**), as determined by flow cytometry after 24 h treatment with the compounds or vehicle alone (control). The percentage of cells in the different phases of the cycle or of apoptosis was calculated by Expo32 software. Values are the mean ± SD of three separate experiments in triplicate.

We next determined alterations in the cell cycle caused by derivatives **1k** and **4c** in colorectal cancer cells. Flow cytometry analysis of nuclear DNA content after 24 h treatment of HCT-116 cells is reported in Figure 2A. Drug concentrations were chosen on the basis that they represent values above and below the respective GI_50_ values. Compared to control cells, **1k** caused a dose-dependent accumulation of cells in G2/M phase, paralleled by a reduction in the percentage of cells in the G1 phase and by a significant increase of cells in the sub-G1 phase, which is representative of cells with fragmented DNA. On the other hand, **4c** induced a dose-dependent accumulation of cells in G1 phase accompanied by a decrement in the percentage of cells in G2/M phases. Moreover, accumulation of sub-G1 population was significantly higher than control (*p* < 0.05) only at high concentration of the drug. These results indicated that the two nortopsentin analogues caused arrest of the HCT-116 cancer cell growth involving different check points of the cell cycle.

#### 2.2.2. Cell Death

To determine whether HCT-116 cells undergo apoptosis upon treatment with the nortopsentin analogues, cells were treated with **1k** or **4c** for 24 h, stained with both propidium iodide (PI) and Annexin V-fluorescein isothiocyanate (FITC), and analyzed by flow cytometry.

Neither compound caused cell necrosis (Figure 2B). Rather, while the percentage of cells in late apoptosis increased at the increase of the **1k** doses, apoptotic effects of **4c** were evident only at high concentrations (GI_70_), when cells in early apoptosis appeared significantly increased with respect to control (*p* < 0.05).

Mitochondria play a critical role in regulating the apoptotic machinery. We then examined mitochondrial membrane potential (Δψm) loss using DiOC6, a fluorescent mitochondria-specific and voltage-dependent dye.

**Figure 3:**
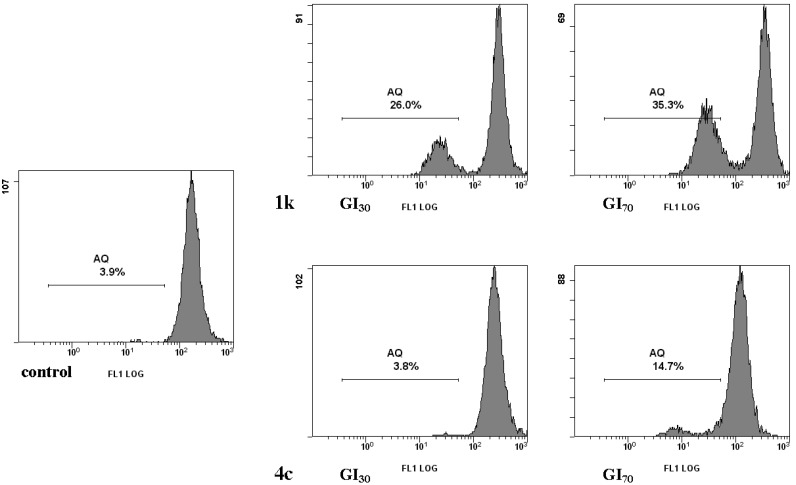
Effects of nortopsentin analogues 1k and 4c on mitochondrial transmembrane potential in HCT-116 cells. The Δψm was detected by fluorescence intensity of 3,30-dihexyloxacarbocyanine iodide-treated cells, as determined by flow cytometry. Control, cells treated with vehicle. Representative images of three experiments with comparable results.

As indicated by the decrement in DiOC6 green-associated fluorescence, treatment of HCT-116 cells with **1k**, for 24 h, induced a remarkable dose-dependent dissipation of Δψm (Figure 3).

On the other hand, incubation with **4c** did not cause mitochondrial dysfunction at GI_30_, whereas induced a significant increase in Δψm loss at higher concentrations.

**Figure 4:**
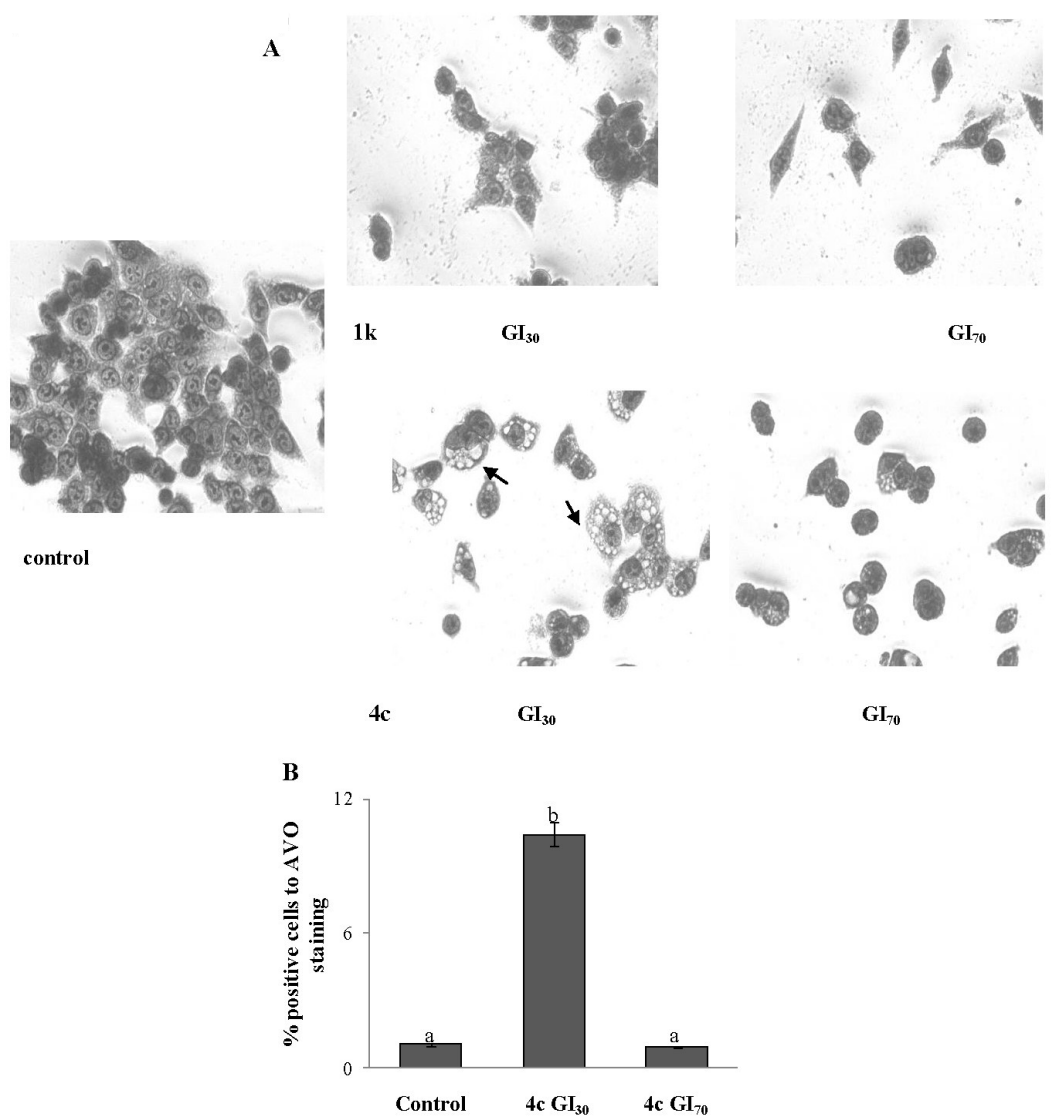
Nortopsentin analogue **4c** induces accumulation of acid vacuoles in HCT-116 cells. (**A**) Micrographs of Giemsa-stained cells in 40× magnification. Control, cells treated with vehicle. Representative images of three experiments with comparable results; (**B**) The percentage of bright red (FL3) fluorescence-positive cells after Acridine Orange (AO) staining and fluorescence-activated cell sorting (FACS) analysis. Results are the mean ± SD of two triplicate experiments. Mean values with unlike superscript letters were significantly different (*p* < 0.05; Bonferroni’s test).

Overall our findings indicated that, although the nortopsentin derivatives inhibited the HCT-116 tumor cell growth, they may elicit different molecular pathways of programmed cell death. Morphology of HTC-116 cells treated for 24 h with nortopsentin analogues **1k** and **4c** was assessed by microscopy analysis after Giemsa staining. Both compounds caused an evident dose-dependent reduction of the cell population with respect to control (Figure 4A). However, whereas **1k** caused highly condensate cells, as a sign of their apoptotic fate, more complex alterations were observed after treatment with **4c**. A low concentration of drug (GI_30_) caused evident expansion of whole cytoplasm, with a massive accumulation of multiple-membrane bounded vacuoles without apparent loss of nuclear material, a morphology characteristic of autophagic cell death. On the other hand, when HCT-116 cells were treated with higher concentration of **4c** (GI_70_), vacuolization was less evident and cells with condensed morphology prevailed, indicating evolution of the cell fate towards apoptosis. Then, we performed FACS analysis of acridine-orange stained acid vacuoles (AVO) using the red to green ratio as an indicator of acid vacuolar organelle accumulation [52]. As shown in Figure 4B, treatment of the cells with **4c** (GI_30_), for 24 h, led to a 10-fold increase of the bright red to green fluorescence intensity ratio of acrydine orange, indicating an elevated induction of cell vacuolization associated to autophagy. Instead, the treatment of cells with the highest drug concentration did not significantly modify the percent of cells with AVO with respect to control, supporting the concept of a different cell death.

A comparison of the antiproliferative activity of the natural products nortopsentins A–C with **1k** and **4c**, the most active derivatives of the two new series of analogues, cannot be performed since nortopsentins were tested against only one cell line (murine leukemia P388), whereas, compounds **1k** and **4c** showed to be active against the 89% and 100% respectively, of the tested cell lines belonging to the NCI full panel of human cancer cell lines (*ca*. 60) with GI_50_ values in the low micromolar range.

A comparison of the antiproliferative activity of **1k** and **4c** with the series of nortopsentin analogues previously reported reveals that these compounds showed to be active against a very large range of human cell lines at the same level of both the analogues in which the central heterocycle was replaced by thiophene, pyrazole, furan, isoxazole, and pyrrole [33,34,35,36,37,38,39]; and those series in which also the indole units were substituted by azaindole systems [42,43,44].

The feature that makes these series of analogues interesting was revealed by the investigations into the cell cycle alterations and cell death modes which suggest that these derivatives inhibiting the HCT-116 tumor cell growth may elicit different molecular pathways of programmed cell death. In fact, 1k induced apoptosis, and 4c exhibited different responses depending on the dose: low concentrations induced morphological changes typical of autophagic death with extensive formation of cytoplasmic acid vacuoles without apparent loss of nuclear material, and with arrest of cell cycle at the G1 phase, instead, higher concentrations induced apoptosis with arrest of cell cycle at the G1 phase.

## 3. Experimental Section

### 3.1. Chemistry

#### 3.1.1. General

All melting point were taken on a Büchi-Tottoly capillary apparatus and are uncorrected. IR spectra were determined in bromoform with a Shimadzu FT/IR 8400S spectrophotometer. ^1^H and ^13^C NMR spectra were measured at 200 and 50.0 MHz, respectively, in DMSO-*d_6_* or CDCl_3_ solution, using a Bruker Avance II series 200 MHz spectrometer. Compounds **2d–f**,**h**,**j**, and **3e**,**f** were characterized only by ^1^H NMR spectra, because of their poor solubility the ^13^C spectra were not performed. Column chromatography was performed with Merk silica gel 230–400 mesh ASTM or with Büchi Sepacor chromatography module (prepacked cartridge system). Elemental analyses (C, H, N) were within ±0.4% of theoretical values and were performed with a VARIO EL III elemental analyzer. Purity of all the tested compounds was greater than 98%, determined by HPLC (Agilent 1100 Series).

#### 3.1.2. Synthesis of 1-Methyl-1*H*-pyrrolo[2,3-*b*]pyridine-3-carbonitrile (**5b**)

To a cold solution of **5a** (1.0 g, 7.0 mmol) in anhydrous toluene (20 mL), *t*-BuOK (1.1 g, 9.5 mmol) and TDA-1 (1 or 2 drops) were added at 0 °C. The reaction mixture was stirred at room temperature for 6 h and then MeI (7.0 mmol, 0.44 mL) was added at 0 °C. TLC analysis (DCM/ethyl acetate 9/1) revealed that methylation was complete after 1 h. The solvent was evaporated under reduced pressure. The residue was treated with water, extracted with DCM (3 × 20 mL), dried (Na_2_SO_4_), evaporated and purified by column chromatography using DCM/ethyl acetate (9/1) as eluent. White solid; yield 85%; mp: 106–107 °C; IR 2224 (CN) cm^−1^; ^1^H NMR (200 MHz, DMSO-*d_6_*) δ: 3.90 (s, 3H, CH_3_), 7.35 (dd, 1H, *J* = 4.7, 8.0 Hz, H-5), 8.15 (dd, 1H, *J* = 1.5, 8.0 Hz, H-4), 8.47 (dd, 1H, *J* = 1.5, 4.7 Hz, H.6), 8.48 (s, 1H, H-2); ^13^C NMR (50 MHz, DMSO-*d_6_*) δ: 31.6 (q), 81.9 (s), 115.3 (s), 118.1 (d), 119.2 (s), 127.6 (d), 138.4 (d), 144.8 (d), 146.4 (s). Anal. Calcd for C_9_H_7_N_3_: C, 68.78; H, 4.49; N, 26.74. Found: C, 68.61; H, 4.21; N, 26.92.

#### 3.1.3. General Procedure for the Synthesis of 1*H*-Pyrrolo[2,3-*b*]pyridine-3-carboxamides (**6a**,**b**)

A solution of appropriate carbonitriles **5a**,**b** (6.3 mmol) in concentrate sulphuric acid (3.32 mL) was stirred at room temperature for 15–60 min. The solution was slowly poured into ice and basified with concentrated NaOH. The residue was extracted with ethyl acetate (3 × 20 mL), dried (Na_2_SO_4_) and concentrated under reduced pressure to afford the carboxamides **6a**,**b**.

##### 1*H*-Pyrrolo[2,3-*b*]pyridine-3-carboxamide (**6a**)

Conditions: room temperature for 60 min. Brown solid; yield: 95%; mp: 273–274 °C; IR 3389, 3335 (NH_2_), 3021 (NH), 1636 (CO) cm^−1^; ^1^H NMR (200 MHz, DMSO-*d_6_*) δ: 7.16 (dd, 1H, *J* = 4.7, 7.9 Hz, H-5), 7.52 (bs, 2H, NH_2_), 8.16 (d, 1H, *J* = 2.5 Hz, H-2), 8.27 (dd, 1H, *J* = 1.7, 4.7 Hz, H-6), 8.46 (dd, 1H, *J* = 1.7, 7.9 Hz, H-4), 12.10 (bs, 1H, NH); ^13^C NMR (50 MHz, DMSO-*d_6_*) δ: 109.3 (s), 116.8 (d), 118.5 (s), 128.6 (d), 129.2 (d), 143.3 (d), 148.4 (s), 165.8 (s). Anal. Calcd for C_8_H_7_N_3_O: C, 59.62; H, 4.38; N, 26.07. Found: C, 59.46; H, 4.59; N, 26.25.

##### 1-Methyl-1*H*-pyrrolo[2,3-*b*]pyridine-3-carboxamide (**6b**)

Conditions: room temperature for 15 min. White solid; yield: 99%; mp: 218–219 °C; IR 3347, 3326 (NH_2_), 1614 (CO) cm^−1^; ^1^H NMR (200 MHz, DMSO-*d_6_*) δ: 3.85 (s, 3H, CH_3_), 7.20 (dd, 1H, *J* = 4.7, 7.9 Hz, H-5), 7.48 (bs, 2H, NH_2_), 8.17 (s, 1H, H-2), 8.33 (dd, 1H, *J* = 1.7, 4.7 Hz, H-6), 8.45 (dd, 1H, *J* = 1.7, 7.9 Hz, H-4); ^13^C NMR (50 MHz, DMSO-*d_6_*) δ: 31.2 (q), 108.2 (s), 117.0 (d), 118.7 (s), 129.4 (d), 132.3 (d), 143.2 (d), 147.4 (s), 165.5 (s). Anal. Calcd for C_9_H_9_N_3_O: C, 61.70; H, 5.18; N, 23.99. Found: C, 61.55; H, 5.44; N, 24.19.

#### 3.1.4. General Procedure for the Synthesis of 1*H*-Pyrrolo[2,3-*b*]pyridine-3-carbothioamides (**7a**,**b**)

A mixture of Lawesson’s reagent (0.33 g, 0.8 mmol) and suitable carboxamides **6a**,**b** (1.3 mmol) in THF (10 mL) was heated under reflux for 30 min. The solution was cooled to room temperature and the solvent was removed under reduced pressure. The residue was purified by column chromatography using ethyl acetate as eluent to give the carbothioamides **7a**,**b**.

##### 1*H*-Pyrrolo[2,3-*b*]pyridine-3-carbothioamide (**7a**)

Yellow solid; yield: 88%; mp: 220–222 °C; IR 3306, 3180 (NH_2_), 3020 (NH), 1590 (CS); ^1^H NMR (200 MHz, DMSO-*d_6_*) δ: 7.22 (dd, 1H, *J* = 4.7, 8.0 Hz, H-5), 8.23 (d, 1H, *J* = 2.7 Hz, H-2), 8.29 (dd, 1H, *J* = 1.5, 4.7 Hz, H-6), 8.99 (dd, 1H, *J* = 1.5, 8.0 Hz, H-4), 9.00 (s, 1H, SH), 9.10 (s, 1H, NH), 12.30 (bs, 1H, NH); ^13^C NMR (50 MHz, DMSO-*d_6_*) δ: 114.7 (s), 117.1 (d), 118.8 (s), 127.6 (d), 130.3 (d), 143.5 (d), 148.9 (s), 193.0 (s). Anal. Calcd for C_8_H_7_N_3_S: C, 54.22; H, 3.98; N, 23.71. Found: C, 54.02; H, 4.08; N, 23.91.

##### 1-Methyl-1*H*-Pyrrolo[2,3-*b*]pyridine-3-carbothioamide (**7b**)

Yellow solid; yield: 99%; mp: 232–233 °C; IR 3336, 3182 (NH_2_), 1540 (CS) cm^−1^; ^1^H NMR (200 MHz, DMSO-*d_6_*) δ: 3.86 (s, 3H, CH_3_), 7.26 (dd, 1H, *J* = 4.7, 8.0 Hz, H-5), 8.28 (s, 1H, H-2), 8.33 (dd, 1H, *J* = 1.6, 4.7 Hz, H-6), 8.93 (dd, 1H, *J* = 1.6, 8.0 Hz, H-4), 8.97 (s, 1H, SH), 9.16 (bs, 1H, NH); ^13^C NMR (50 MHz, DMSO-*d_6_*) δ: 31.3 (q), 113.7 (s), 117.4 (d), 118.7 (s), 130.3 (d), 131.8 (d), 143.3 (d), 147.8 (s), 192.6 (s). Anal. Calcd for C_9_H_9_N_3_S: C, 56.52; H, 4.74; N, 21.97. Found: C, 56.32; H, 4.59; N, 22.27.

#### 3.1.5. General Procedure for the Synthesis of 1-Methyl-1*H*-pyrrolo[2,3-*b*]pyridines (**8b**,**d**,**f**)

To a cold solution of appropriate pyrrolo-pyridines **8a**,**c**,**e** (2.5 mmol) in anhydrous toluene (25 mL), *t*-BuOK (0.38 g, 3.4 mmol) and TDA-1 (1 or 2 drops) were added at 0 °C. The reaction mixture was stirred at room temperature for 3 h and then MeI (2.5 mmol, 0.2 mL) was added at 0 °C. TLC analysis (ethyl acetate) revealed that methylation was complete after 1 h. The solvent was evaporated under reduced pressure. The residue was treated with water, extracted with DCM (3 × 20 mL), dried (Na_2_SO_4_), evaporated and purified by column chromatography using DCM/ethyl acetate (9/1) as eluent to give derivatives **8b**,**d**,**f** [42].

##### 1-Methyl-1*H*-pyrrolo[2,3-*b*]pyridine (**8b**)

Oil; yield: 96%; Analytical and spectroscopic data were previously reported [42].

##### 5-Bromo-1-methyl-1*H*-pyrrolo[2,3-*b*]pyridine (**8d**)

Brown solid; yield: 85%; mp: 62–63 °C; ^1^H NMR (200 MHz, DMSO-*d_6_*) δ: 3.83 (s, 3H, CH_3_), 6.44 (d, 1H, *J* = 3.4 Hz, H-3), 7.60 (d, 1H, *J* = 3.4 Hz, H-2), 8.21 (d, 1H, *J* = 2.1 Hz, H-4), 8.33 (d, 1H, *J* = 2.1 Hz, H-6); ^13^C NMR (50 MHz, DMSO-*d_6_*) δ: 31.0 (q), 98.5 (d), 110.7 (s), 121.7 (s), 130.3 (d), 131.8 (d), 142.2 (d), 145.7 (s). Anal. Calcd for C_8_H_7_BrN_2_: C, 45.53; H, 3.34; N, 13.27. Found: C, 45.38; H, 3.24; N, 13.45.

##### 5-Fluoro-1-methyl-1*H*-pyrrolo[2,3-*b*]pyridine (**8f**)

Brown solid; yield: 60%; mp: 72–74 °C; ^1^H NMR (200 MHz, DMSO-*d_6_*) δ: 3.83 (s, 3H, CH_3_), 6.47 (1H, d, *J* = 3.4 Hz, H-3) 7.63 (d, 1H, *J* = 3.4 Hz, H-2), 7.87 (dd, 1H, *J* = 2.7, 9.5 Hz, H-4), 8.24–8.27 (m, 1H, H-6); ^13^C NMR (50 MHz, DMSO-*d_6_*) δ: 31.1 (q), 98.8 (d, *J_C3-F_* = 4.5 Hz), 113.9 (d, *J_C4-F_* = 20.5 Hz), 119.9 (s, *J_C3a–f_* = 7.5 Hz), 130.4 (d, *J_C6-F_* = 29.2 Hz), 132.3 (d), 144.3 (s), 155.0 (s, *J_C5-F_* = 238.0 Hz). Anal. Calcd for C_8_H_7_FN_2_: C, 63.99; H, 4.70; N, 18.66. Found: C, 63.69; H, 4.91; N, 18.48.

#### 3.1.6. General Procedure for the Synthesis of 2-Bromo-1-(1*H*-pyrrolo[2,3-*b*]pyridin-3-yl)ethanones (**9a–f**)

To a solution of appropriate pyrrolo-pyridines **8a–f** (2.5 mmol) in 10 mL of anhydrous DCM, anhydrous AlCl_3_ (1.2 g, 8.8 mmol) was slowly added. The reaction mixture was heated under reflux and BrCOCH_2_Br (2.5 mmol, 0.2 mL) in 2 mL of anhydrous DCM was added dropwise. The resulting solution was allowed to stir under reflux for 40 min. After cooling, water and ice were slowly added and the obtained precipitate (for derivative **9a**) was filtered off or the oil residue (for derivatives **9b–f**) was extracted with DCM (3 × 20 mL) and purified by column chromatography using DCM/ethyl acetate (9/1) as eluent [42].

##### 2-Bromo-1-(1*H*-pyrrolo[2,3-*b*]pyridin-3-yl)pthanone (**9a**)

White solid; yield: 92%; mp: 280–282 °C; Analytical and spectroscopic data were previously reported [42].

##### 2-Bromo-1-(1-methyl-1*H*-pyrrolo[2,3-*b*]pyridin-3-yl)ethanone (**9b**)

White solid; yield: 80%; mp: 116–117 °C; Analytical and spectroscopic data were previously reported [42].

##### 2-Bromo-1-(5-bromo-1*H*-pyrrolo[2,3-*b*]pyridin-3-yl)ethanone (**9c**)

Brown solid; yield: 75%; mp: 244–245 °C; IR 3019 (NH), 1655 (CO) cm^−1^; ^1^H NMR (200 MHz, DMSO-*d_6_*) δ: 4.72 (s, 2H, CH_2_), 8.46 (d, 1H, *J* = 2.2 Hz, H-4), 8.57 (d, 1H, *J* = 2.2 Hz, H-6), 8.71 (d, 1H, *J* = 3.2 Hz, H-2), 13.00 (bs, 1H, NH); ^13^C NMR (50 MHz, DMSO-*d_6_*) δ: 33.1 (t), 111.7 (s), 113.8 (s), 119.4 (s), 131.2 (d), 136.9 (d), 144.8 (d), 147.4 (s), 186.6 (s). Anal. Calcd for C_9_H_6_Br_2_N_2_O: C, 34.00; H, 1.90; N, 8.81. Found: C, 33.87; H, 2.11; N, 8.99.

##### 2-Bromo-1-(5-bromo-1-methyl-1*H*-pyrrolo[2,3-*b*]pyridin-3-yl)ethanone (**9d**)

Brown solid; yield: 70%; mp: 166–167 °C; IR 1653 (CO) cm^−1^; ^1^H NMR (200 MHz, DMSO-*d_6_*) δ: 3.89 (s, 3H, CH_3_), 4.66 (s, 2H, CH_2_), 8.49 (d, 1H, *J* = 2.2 Hz, H-4), 8.54 (d, 1H, *J* = 2.2 Hz, H-6), 8.74 (s, 1H, H-2); ^13^C NMR (50 MHz, DMSO-*d_6_*) δ: 31.9 (q), 32.9 (t), 110.2 (s), 114.2 (s), 119.6 (s), 131.4 (d), 139.9 (d), 144.6 (d), 146.5 (s), 186.1 (s). Anal. Calcd for C_10_H_8_Br_2_N_2_O: C, 36.18; H, 2.43; N, 8.44. Found: C, 36.02; H, 2.67; N, 8.64.

##### 2-Bromo-1-(5-fluoro-1*H*-pyrrolo[2,3-*b*]pyridin-3-yl)ethanone (**9e**)

Brown solid; yield: 74%; mp: 206–207 °C; IR 3555 (NH), 1654 (CO) cm^−1^; ^1^H NMR (200 MHz, DMSO-* d_6_*) δ: 4.71 (s, 2H, CH_2_), 8.20 (dd, 1H, *J* = 2.8, 9.1 Hz, H-4), 8.37–8.39 (m, 1H, H-6), 8.73 (d, 1H, *J* = 3.3 Hz, H-2), 12.88 (bs, 1H, NH); ^13^C NMR (50 MHz, DMSO-*d_6_*) δ: 33.7 (t), 112.2 (s, *J_C7a–f_* = 3.9 Hz), 115.0 (d, *J_C4-F_* = 21.5 Hz), 118.1 (s, *J_C3a–f_* = 7.9 Hz), 133.0 (d, *J_C6-F_* = 26.5 Hz), 137.4 (d), 145.7 (s), 156.4 (s, *J_C5-F_* = 243.0 Hz), 186.5 (s). Anal. Calcd for C_9_H_6_BrFN_2_O: C, 42.05; H, 2.35; N, 10.90. Found: C, 41.85; H, 2.56; N, 10.76.

##### 2-Bromo-1-(5-fluoro-1-methyl-1*H*-pyrrolo[2,3-*b*]pyridin-3-yl)ethanone (**9f**)

Brown solid; yield: 74%; mp: 149–150 °C; IR 1668 (CO) cm^−1^; ^1^H NMR (200 MHz, DMSO-* d_6_*) δ: 3.90 (s, 3H, CH_3_), 4.65 (s, 2H, CH_2_), 8.20 (dd, 1H, *J* = 2.8, 9.0 Hz, H-4), 8.42–8.44 (m, 1H, H-6), 8.78 (s, 1H, H-2); ^13^C NMR (50 MHz, DMSO-*d_6_*) δ: 32.0 (q), 32.8 (t), 110.6 (s, *J_C7a–f_* = 3.9 Hz), 115.3 (d, *J_C4-F_* = 21.9 Hz), 118.3 (s, *J_C3a–f_* = 7.9 Hz), 132.8 (d, *J_C6-F_* = 29.3 Hz), 140.4 (d), 144.8 (s), 156.7 (s, *J_C5-F_* = 243.5 Hz), 186.0 (s). Anal. Calcd for C_10_H_8_BrFN_2_O: C, 44.31; H, 2.97; N, 10.33. Found: C, 44.14; H, 2.73; N, 10.53.

#### 3.1.7. General Procedure for the Synthesis of Substituted-(1,3-thiazole-2,4-diyl)-bis(1*H*-pyrrolo[2,3-*b*]Pyridines (**1a–l**)

A suspension of the appropriate pyrrolo[2,3-*b*]pyridine-carbothioamides **7a**,**b** (5 mmol) and the proper 3-bromo-acetyl**-**pyrrolo[2,3-*b*]pyridines **9a–f** (5 mmol) in anhydrous EtOH (20 mL) was heated under reflux for 30 min. The precipitate, obtained after cooling, was filtered off, dried and crystallized with EtOH to afford derivatives **1a–l**.

##### 3,3′-(1,3-Thiazole-2,4-diyl)bis(1*H*-pyrrolo[2,3-*b*]pyridine) (**1a**)

Yellow solid; yield: 63%; mp: 346–347 °C; IR 3410, 3166 (NH) cm^−1^; ^1^H NMR (200 MHz, DMSO-*d_6_*) δ: 7.48 (dd, 1H, *J* = 5.0, 7.9 Hz, ArH), 7.60 (dd, 1H, *J* = 5.5, 7.9 Hz, ArH), 8.00 (s, 1H, Ar), 8.39 (s, 1H, ArH), 8.46 (s, 1H, ArH), 8.48 (d, 1H, *J* = 5.5 Hz, ArH), 8.55 (d, 1H, *J* = 5.0 Hz, ArH), 8.87 (d, 1H, *J* = 7.9 Hz, ArH), 9.11 (d, 1H, *J* = 7.9 Hz, ArH), 12.75 (bs, 1H, NH), 12.90 (bs, 1H, NH). ^13^C NMR (50 MHz, DMSO*-d_6_*) δ: 109.6 (d), 110.5 (s), 116.3 (d), 117.3 (d), 118.7 (s), 125.8 (s), 126.9 (d), 127.9 (d), 129.6 (s), 131.4 (d), 134.8 (d), 137.2 (d), 141.5 (d), 143.2 (s), 145.9 (s), 147.9 (s), 149.2 (s). Anal. Calcd for C_17_H_11_N_5_S: C, 64.34; H, 3.49; N, 22.07. Found: C, 64.54; H, 3.70; N, 22.23.

##### 5-Bromo-3-[2-(1*H*-pyrrolo[2,3-*b*]pyridin-3-yl)-1,3-thiazol-4-yl)-1*H*-pyrrolo[2,3-*b*]pyridine (**1b**)

Yellow solid; yield: 60%; mp: 390–391 °C; IR 3334, 3350 (NH) cm^−1^; ^1^H NMR (200 MHz, DMSO*-d_6_*) δ: 7.35 (dd, 1H, *J* = 4.8, 8.0 Hz, H-5′), 7.87 (s, 1H, H-5), 8.24 (d, 1H, *J* = 2.6 Hz, H-2″), 8.35 (d, 1H, *J* = 2.1 Hz, H-6″), 8.38–8.42 (m, 2H, H-2′, H-6′), 8.80 (dd, 1H, *J* = 1.2, 8.0 Hz, H-4′), 8.81 (d, 1H, *J* = 2.1 Hz, H-4″), 12.26 (bs, 1H, NH), 12.46 (bs, 1H, NH); ^13^C NMR (50 MHz, DMSO*-d_6_*) δ: 108.2 (d), 109.5 (s), 109.8 (s), 111.5 (s), 117.1 (d), 118.3 (s), 118.7 (s), 126.8 (d), 127.5 (d), 130.3 (d), 130.9 (d), 131.6 (s), 142.0 (d), 143.0 (d), 147.0 (s), 149.0 (s), 161.0 (s). Anal. Calcd for C_17_H_10_BrN_5_S: C, 51.53; H, 2.54; N, 17.67. Found: C, 51.30; H, 2.26; N, 17.86.

##### 5-Fluoro-3-[2-(1*H*-pyrrolo[2,3-*b*]pyridin-3-yl)-1,3-thiazol-4-yl]-1*H*-pyrrolo[2,3-*b*]pyridine (**1c**)

Yellow solid; yield: 75%; mp: 373–374 °C; IR 3247, 3156 (NH) cm^−1^; ^1^H NMR (200 MHz, DMSO*-d_6_*) δ: 7.37 (dd, 1H, *J* = 4.8, 7.9 Hz, H-5′), 7.83 (s, 1H, H-5), 8.27 (d, 1H, *J* = 2.6 Hz, H-2″), 8.31 (t, 1H, *J* = 1.7 Hz, H-6″), 8.36 (d, 1H, *J* = 2.2 Hz, H-2′), 8.41 (dd, 1H, *J* = 1.3, 4.8 Hz, H-6′), 8.48 (d, 1H, *J* = 1.7 Hz, H-4″), 8.80 (dd, 1H, *J* = 1.3, 7.9 Hz, H-4′), 12.19 (bs, 1H, NH), 12.49 (bs, 1H, NH); ^13^C NMR (50 MHz, DMSO*-d_6_*) δ: 108.1 (d), 110.0 (s), 114.0 (d, *J_C-4”_* = 21.2 Hz), 116.9 (s), 117.1 (s), 117.2 (d), 127.7 (d), 128.1 (d), 131.2 (d, *J_C-6”_* = 28.6 Hz), 132.7 (d), 140.4 (d), 144.8 (s), 145.5 (s), 147.4 (s), 149.2 (s), 153.0 (s), 159.2 (s, *J_C-5”_* = 149 Hz). Anal. Calcd for C_17_H_10_FN_5_S: C, 60.88; H, 3.01; N, 20.88. Found: C, 60.58; H, 2.80; N, 21.04.

##### 1-Methyl-3-[4-(1*H*-pyrrolo[2,3-*b*]pyridin-3-yl)-1,3-thiazol-2-yl]-1*H*-pyrrolo[2,3-*b*]pyridine (**1d**)

Yellow solid; yield: 90%; mp: 278–279 °C; IR 3450 (NH) cm^−1^; ^1^H NMR (200 MHz, DMSO*-d_6_*) δ: 3.95 (s, 3H, CH_3_), 7.39 (dd, 1H, *J* = 4.8, 7.9 Hz, ArH), 7.55 (dd, 1H, *J* = 5.4, 7.8 Hz, ArH), 7.93 (s, 1H, ArH), 8.33 (s, 1H, ArH), 8.44–8.53 (m, 3H, 3xArH), 8.69 (d, 1H, *J* = 7.9 Hz, ArH), 9.05 (d, 1H, *J* = 7.8 Hz, ArH), 12.80 (bs, 1H, NH); ^13^C NMR (50 MHz, DMSO*-d_6_*) δ: 31.4 (q), 107.7 (s), 109.0 (d), 110.7 (s), 115.8 (s), 116.2 (d), 117.3 (d), 120.0 (s), 126.6 (d), 129.4 (d), 130.7 (d), 134.18 (d), 137.7 (d), 142.4 (s), 143.2 (d), 146.7 (s), 148.1 (s), 151.0 (s). Anal. Calcd for C_18_H_13_N_5_S: C, 65.24; H, 3.95; N, 21.13. Found: C, 65.06; H, 4.19; N, 21.43.

##### 3-[4-(5-Bromo-1*H*-pyrrolo[2,3-*b*]pyridin-3-yl)-1,3-thiazol-2-yl]-1-methyl-1*H*-pyrrolo[2,3-*b*]pyridine (**1e**)

Yellow solid; yield: 94%; mp: 311 °C; IR 3347 (NH) cm^−1^; ^1^H NMR (200 MHz, DMSO*-d_6_*) δ: 3.94 (s, 3H, CH_3_), 7.36 (dd, 1H, *J* = 4.7, 7.9 Hz, H-5′), 7.86 (s, 1H, H-5), 8.23 (d, 1H, *J* = 2.6 Hz, H-2″), 8.39 (d, 1H, *J* = 2.2 Hz, H-6″), 8.41 (s, 1H, H-2′), 8.44 (dd, 1H, *J* = 1.6, 4.7 Hz, H-6′), 8.72 (dd, 1H, *J* = 1.6, 7.9 Hz, H-4′), 8.81 (d, 1H, *J* = 2.2 Hz, H-4″), 12.27 (bs, 1H, NH); ^13^C NMR (50 MHz, DMSO*-d_6_*) δ: 31.6 (q), 108.1 (d), 108.3 (s), 109.4 (s), 111.5 (s), 117.2 (d), 117.6 (s), 118.8 (s), 126.8 (d), 130.2 (d), 130.4 (d), 130.8 (d), 142.6 (d), 142.9 (d), 146.5 (s), 147.0 (s), 149.0 (s), 161.0 (s). Anal. Calcd for C_18_H_12_BrN_5_S: C, 52.69; H, 2.95; N, 17.07. Found: C, 52.84; H, 3.19; N, 16.93.

##### 3-[4-(5-Fluoro-1*H*-pyrrolo[2,3-*b*]pyridin-3-yl)-1,3-thiazol-2-yl]-1-methyl-1*H*-pyrrolo[2,3-*b*]pyridine (**1f**)

Yellow solid; yield: 90%; mp: 316 °C; IR 3377 (NH) cm^−1^; ^1^H NMR (200 MHz, DMSO*-d_6_*) δ: 3.94 (s, 3H, CH_3_), 7.38 (dd, 1H, *J* = 4.8, 7.9 Hz, H-5′), 7.83 (s, 1H, H-5), 8.27 (d, 1H, *J* = 2.7 Hz, H-2″), 8.32 (t, 1H, *J* = 1.7 Hz, H-6″), 8.43–8.48 (m, 3H, H-2′, H-4″, H-6′), 8.71 (dd, 1H, *J* = 1.5, 7.9 Hz, H-4′), 12.18 (bs, 1H, NH). ^13^C NMR (50 MHz, DMSO*-d_6_*) δ: 31.5 (q), 107.6 (d), 108.2 (s), 109.9 (s, *J_C7″a–f_* = 4.2 Hz), 114.1 (d, *J_C4″-F_* = 21.3 Hz), 117.0 (s, *J_C3″a–f_* = 7.27 Hz), 117.3 (d), 117.6 (s), 127.5 (d), 127.6 (d), 130.4 (d, *J_C6″-F_* = 27.2 Hz), 130.9 (d), 131.5 (d), 142.7 (d), 145.5 (s), 146.6 (s), 149.2 (s), 155.3 (s, *J_C5″-F_* = 239 Hz), 160.9 (s). Anal. Calcd for C_18_H_12_FN_5_S: C, 61.88; H, 3.46; N, 20.04. Found: C, 61.68; H, 3.72; N, 20.23.

##### 3,3′-(1,3-Thiazole-2,4-diyl)bis(1-methyl-1*H*-pyrrolo[2,3-*b*]pyridine) (**1g**)

Yellow solid; yield: 60%; mp: 310–311 °C; ^1^H NMR (200 MHz, DMSO*-d_6_*) δ: 3.94 (s, 3H, CH_3_), 3.98 (s, 3H, CH_3_), 7.36–7.45 (m, 2H, 2xArH), 7.82 (s, 1H, ArH), 8.32 (s, 1H, ArH), 8.42 (s, 1H, ArH), 8.45–8.52 (m, 2H, 2xArH), 7.75 (dd, 1H, *J* = 1.0, 7.9 Hz, ArH), 8.81 (d, 1H, *J* = 7.8 Hz, ArH); ^13^C NMR (50 MHz, DMSO*-d_6_*) δ: 31.4 (q), 31.7 (q), 108.2 (d), 109.5 (s), 116.1 (d), 117.3 (d), 117.4 (s), 129.9 (d), 130.8 (d), 131.5 (d), 140.0 (d), 140.1 (d), 143.0 (d), 144.9 (s), 145.1 (s), 145.2 (s), 146.9 (s), 148.7 (s), 161.2 (s). Anal. Calcd for C_19_H_15_N_5_S: C, 66.07; H, 4.38; N, 20.27. Found: C, 65.92; H, 4.20; N, 20.40.

##### 5-Bromo-1-methyl-3-[2-(1-methyl-1*H*-pyrrolo[2,3-*b*]pyridin-3-yl)-1,3-thiazol-4-yl]-1*H* pyrrolo[2,3-*b*]pyridine (**1h**)

Yellow solid; yield: 90%; mp: 308 °C; ^1^H NMR (200 MHz, DMSO*-d_6_*) δ: 3.92 (s, 3H, CH_3_), 3.93 (s, 3H, CH_3_), 7.36 (dd, 1H, *J* = 4.7, 7.9 Hz, H-5′), 7.85 (s, 1H, H-5), 8.31 (s, 1H, H-2″), 8.40 (s, 1H, H-2′), 8.42 (dd, 1H, *J* = 1.6, 4.7 Hz, H-6′), 8.45 (d, 1H, *J* = 2.2 Hz, H-6″), 8.73 (dd, 1H, *J* = 1.6, 7.9 Hz, H-4′), 8.79 (d, 1H, *J* = 2.2 Hz, H-4″); ^13^C NMR (50 MHz, DMSO*-d_6_*) δ: 31.2 (q), 31.5 (q), 107.9 (d), 108.2 (s), 108.3 (s), 111.7 (s), 117.2 (d), 117.5 (s), 118.7 (s), 130.0 (d), 130.4 (d), 130.5 (d), 130.6 (d), 130.7 (d), 142.9 (d), 146.0 (s), 146.7 (s), 148.6 (s), 161.0 (s). Anal Calcd for C_19_H_14_BrN_5_S: C, 53.78; H, 3.33; N, 16.50. Found: C, 53.65; H, 3.08; N, 16.73.

##### 5-Fluoro-1-methyl-3-[2-(1-methyl-1*H*-pyrrolo[2,3-*b*]pyridin-3-yl)-1,3-thiazol-4-yl]-1*H*-pyrrolo[2,3-*b*]pyridine (**1i**)

Yellow solid; yield: 60%; mp: 301–302 °C; ^1^H NMR (200 MHz, DMSO*-d_6_*) δ: 3.92 (s, 3H, CH_3_), 3.93 (s, 3H, CH_3_), 7.37 (dd, 1H, *J* = 4.7, 7.9 Hz, H-5′), 7.81 (s, 1H, H-5), 8.35–8.49 (m, 5H, H-2′, H-2″, H4″, H-6′, H-6″), 8.72 (dd, 1H, *J* = 1.5, 7.9 Hz, H-4′); ^13^C NMR (50 MHz, DMSO*-d_6_*) δ: 31.3 (q), 31.4 (q), 107.4 (d), 108.2 (s), 108.7 (s, *J_C7″a–f_* = 4.2 Hz), 114.2 (d, *J_C4″-F_* = 21.7 Hz), 117.0 (s, *J_C3″a–f_* = 7.3 Hz), 117.2 (d), 117.4 (s), 130.0 (d), 130.8 (d), 131.0 (d, *J_C6″-F_* = 27.1 Hz), 131.4 (d), 143.0 (d), 144.5 (s), 146.9 (s), 148.9 (s), 153.2 (s), 159.5 (s, *J_C5″-F_* = 155.0 Hz). Anal. Calcd for C_19_H_14_FN_5_S: C, 62.79; H, 3.88; N, 19.27. Found: C, 62.94; H, 4.15; N, 19.38.

##### 1-Methyl-3-[4-(1*H*-pyrrolo[2,3-*b*]pyridin-3-yl)-1,3-thiazol-4-yl]-1*H*-pyrrolo[2,3-*b*]pyridine (**1j**)

Yellow solid; yield: 85%; mp: 303–304 °C; IR 3410 (NH) cm^−1^; ^1^H NMR (200 MHz, DMSO*-d_6_*) δ: 3.96 (s, 3H, CH_3_), 7.36 (dd, 1H, *J* = 4.9, 7.9 Hz, ArH), 7.42 (dd, 1H, *J* = 4.9, 7.9 Hz, ArH), 7.82 (s, 1H, ArH), 8.31 (s, 1H, ArH), 8.39 (s, 1H, ArH), 8.42–8.46 (m, 2H, 2xArH), 8.73 (dd, 1H, *J* = 1.5, 7.9 Hz, ArH), 8.87 (dd, 1H, *J* = 1.5, 7.9 Hz, ArH), 12.60 (bs, 1H, NH); ^13^C NMR (50 MHz, DMSO-*d_6_*) δ: 31.44 (q), 108.0 (d), 109.3 (s), 109.8 (s), 116.2 (d), 117.2 (d), 118.2 (s), 118.5 (s), 127.5 (d), 129.6 (d), 130.2 (d), 130.8 (d), 131.5 (s), 140.7 (s), 141.4 (d), 141.6 (s), 142.3 (d), 148.9 (s). Anal. Calcd for C_18_H_13_N_5_S: C, 65.24; H, 3.95; N, 21.13. Found: C, 65.09; H, 3.69; N, 21.36.

##### 5-Bromo-1-methyl-3-[2-(1*H*-pyrrolo[2,3-*b*]pyridin-3-yl)-1,3-thiazol-4-yl]-1*H*-pyrrolo[2,3-*b*]pyridine (**1k**)

Yellow solid; yield: 80%; mp: 301 °C; IR 3174 (NH) cm^−1^; ^1^H NMR (200 MHz, DMSO*-d_6_*) δ: 3.92 (s, 3H, CH_3_), 7.35 (dd, 1H, *J* = 4.8, 7.9 Hz, H-5′), 7.86 (s, 1H, H-5), 8.32 (s, 1H, H-2″), 8.33 (d, 1H, *J* = 3.1 Hz, H-2′), 8.41 (dd, 1H, *J* = 1.4, 4.8 Hz, H-6′), 8.43 (d, 1H, *J* = 2.2 Hz, H-6″), 8.79 (dd, 1H, *J* = 1.4, 7.9 Hz, H-4′), 8.80 (d, 1H, *J* = 2.2 Hz, H-4″), 12.44 (bs, 1H, NH); ^13^C NMR (50 MHz, DMSO*-d_6_*) δ: 31.2 (q), 108.4 (d), 109.9 (s), 111.7 (s), 117.1 (d), 118.6 (s), 118.7 (s), 127.9 (d), 130.4 (d), 130.7 (d), 132.1 (d), 141.0 (d), 141.1 (s), 142.9 (d), 145.4 (s), 146.0 (s), 148.6 (s), 160.9 (s). Anal. Calcd for C_18_H_12_BrN_5_S: C, 52.69; H, 2.95; N, 17.07. Found: C, 52.93; H, 3.09; N, 17.23.

##### 5-Fluoro-1-methyl-3-[2-(1*H*-pyrrolo[2,3-*b*]pyridin-3-yl)-1,3-thiazol-4-yl]-1*H*-pyrrolo[2,3-*b*]pyridine (**1l**)

Yellow solid; yield: 72%; mp: 295–296 °C; IR 3434 (NH) cm^−1^; ^1^H NMR (200 MHz, DMSO*-d_6_*) δ: 3.93 (s, 3H, CH_3_), 7.38 (dd, 1H, *J* = 4.8, 7.9 Hz, H-5′), 7.83 (s, 1H, H-5), 8.36–8.50 (m, 5H, H-2′, H-2″, H-4″, H-6′, H-6″), 8.81 (dd, 1H, *J* = 1.4, 7.9 Hz, H-4′), 12.50 (bs, 1H, NH); ^13^C NMR (50 MHz, DMSO-*d_6_*) δ*:* 31.3 (q), 107.8 (d), 108.7 (s, *J_C3a″-F_* = 4.1 Hz), 109.8 (s), 114.2 (d, *J_C6″-F_* = 21.8 Hz), 116.9 (s), 117.1 (d), 117.2 (d), 118.3 (s), 127.7 (d), 130.8 (d), 131.5 (d, *J_C4-F″_* = 14.1 Hz), 141.5 (d), 144.5 (s), 145.9 (s), 148.9 (s), 153.2 (s), 159.4 (s, *J_C5′-F′_* = 152.0 Hz). Anal. Calcd for C_18_H_12_FN_5_S: C, 61.88; H, 3.46; N, 20.04. Found: C, 62.04; H, 3.70; N, 20.19.

#### 3.1.8. Synthesis of 7-Chloro-1-methyl-1*H*-pyrrolo[2,3-*c*]pyridine (**22**)

To a cold solution of 7-chloro-1*H*-pyrrolo[2,3-*c*]pyridine **21** (0.12 g, 0.8 mmol) in anhydrous toluene (4 mL), *t*-BuOK (0.12 g, 1.1 mmol) and TDA-1 (1 or 2 drops) were added at 0 °C. The reaction mixture was stirred at room temperature for 5 h and then MeI (0.8 mmol, 0.05 mL) was added at 0 °C and then stirred room temperature. TLC analysis (DCM/ethyl acetate 9/1) revealed that methylation was complete after 2 h. The solvent was evaporated under reduced pressure. The residue was treated with water, extracted with DCM (3 × 20 mL), dried (Na_2_SO_4_), evaporated and purified by column chromatography using DCM/ethyl acetate (95/5) as eluent. White solid; yield: 70%; mp: 60–61 °C; ^1^H NMR (200 MHz, CDCl_3_) δ: 4.16 (s, 3H, CH_3_), 6.49 (d, 1H, *J* = 3.1 Hz, H-3), 7.16 (d, 1H, *J* = 3.1 Hz, H-2), 7.41 (d, 1H, *J* = 5.4 Hz, H-4), 7.95 (d, 1H, *J* = 5.4 Hz, H-5); ^13^C NMR (50 MHz, CDCl_3_) δ: 36.7 (q), 101.0 (d), 115.2 (d), 129.2 (s), 134.1 (s), 134.7 (d), 136.5 (s), 137.5 (d). Anal. Calcd for: C_8_H_7_ClN_2_: C, 57.67; H, 4.23; N, 16.81. Found: C, 57.90; H, 3.97; N, 16.63.

#### 3.1.9. General Procedure for the Synthesis of 1-(7-Chloro-1*H*-pyrrolo[2,3-*c*]pyridin-3-yl) ethanones (**23**, **24**)

To a solution of appropriate pyrrolo[2,3-*c*]pyridines **21**, **22** (2.5 mmol) in 10 mL of anhydrous DCM, anhydrous AlCl_3_ (1.2 g, 8.8 mmol) was slowly added. The reaction mixture was heated under reflux and BrCOCH_2_Br (2.5 mmol, 0.2 mL) in 2 mL of anhydrous DCM was added dropwise. The resulting solution was allowed to stir under reflux for 40 min. After cooling, water and ice were slowly added and the obtained precipitate (for derivative **23**) was filtered off or the oil residue (for derivative **24**) was extracted with DCM (3 × 20 mL) and purified by column chromatography using DCM/ethyl acetate 9/1 as eluent.

##### 2-Bromo-1-(7-Chloro-1*H*-pyrrolo[2,3-*c*]pyridin-3-yl)ethanone (**23**)

White solid; yield: 88%; mp: 324–325 °C; IR 3557 (NH), 1676 (CO) cm^−1^; ^1^H NMR (200 MHz, DMSO-*d_6_*) δ: 4.76 (s, 2H, CH_2_), 8.05 (d, 1H, *J* = 5.2 Hz, H-4), 8.15 (d, 1H, *J* = 5.2 Hz, H-5), 8.74 (d, 1H, *J* = 3.0 Hz, H-2), 13.03 (bs, 1H, NH); ^13^C NMR (50 MHz, DMSO-*d_6_*) δ: 33.8 (t), 114.2 (s), 115.6 (d), 130.2 (s), 132.7 (s), 134.3 (s), 138.5 (d), 140.6 (d), 186.6 (s). Anal. Calcd for: C_9_H_6_BrClN_2_O: C, 39.52; H, 2.21; N, 10.24. Found: C, 39.28; H, 2.36; N, 10.13.

##### 2-Bromo-1-(7-chloro-1-methyl-1*H*-pyrrolo[2,3-*c*]pyridin-3-yl)ethanone (**24**)

White solid; yield: 90%; mp: 249–250 °C; IR 1647 (CO) cm^−1^; ^1^H NMR (200 MHz, DMSO-*d_6_*) δ: 4.18 (s, 3H, CH_3_), 4.90 (s, 2H, CH_2_), 8.07 (d, 1H, *J* = 5.3 Hz, H-4), 8.12 (d, 1H, *J* = 5.3 Hz, H-5), 8.72 (s, 1H, H-2); ^13^C NMR (50 MHz, DMSO-*d_6_*) δ: 33.3 (t), 37.4 (q), 111.8 (s), 115.7 (d), 129.6 (s), 133.9 (s), 134.3 (s), 140.7 (d), 143.2 (d), 185.9 (s). Anal. Calcd for: C_10_H_8_BrClN_2_O: C, 41.77; H, 2.80; N, 9.74. Found: C, 41.51; H, 3.07; N, 9.56.

#### 3.1.10. General Procedure for the Synthesis of Indolyl-thiazolyl-pyrrolo[2,3-*c*]pyridines (**2a–j**), (**3a–f**)

A suspension of the appropriate indole-3-carbothioamides **16d**, **17a–d**, **18a–c** (5.0 mmol) and the proper 3-bromoacetyl-pyrrolo[2,3-*c*]pyridines **23**, **24** (5.0 mmol) in anhydrous EtOH (20 mL) was heated under reflux for 1–3 h. The precipitate, obtained after cooling, was filtered off, dried and crystallized from EtOH to afford derivatives **2a–j** and **3a–f**.

##### 7-Chloro-3-[2-(1-methyl-1*H*-indol-3-yl)-1,3-thiazol-4-yl]-1*H*-pyrrolo[2,3-*c*]pyridine (**2a**)

Conditions: reflux for 1 h. Yellow solid; yield: 98%; mp: 326–327 °C; IR 3393 (NH) cm^−1^; ^1^H NMR (200 MHz, DMSO-*d_6_*) δ: 3.93 (s, 3H, CH_3_), 7.29–7.35 (m, 2H, H-5′, H-6′), 7.56–7.61 (m, 1H, H-7′), 7.85 (s, 1H, H-2′), 8.12 (d, 1H, *J* = 5.6 Hz, H-4″), 8.26 (s, 1H, H-5), 8.28–8.31 (m, 1H, H-4′), 8.33 (d, 1H, *J* = 5.6 Hz, H-5″), 8.40 (d, 1H, *J* = 2.7 Hz, H-2″), 12.62 (bs, 1H, NH); ^13^C NMR (50 MHz, DMSO-*d_6_*) δ: 32.9 (q), 108.3 (d), 109.2 (s), 110.7 (d), 112.4 (s), 115.3 (d), 120.2 (d), 121.1 (d), 122.5 (d), 124.5 (s), 129.8 (s), 130.5 (d), 130.8 (d), 131.8 (s), 133.4 (s), 136.7 (d), 137.0 (s), 148.1 (s), 162.2 (s). Anal. Calcd for: C_19_H_13_ClN_4_S: C, 62.55; H, 3.59; N, 15.36. Found: C, 62.28; H, 3.34; N, 15.53.

##### 7-Chloro-3-[2-(5-methoxy-1-methyl-1*H*-indol-3-yl)-1,3-thiazol-4-yl]-1*H*-pyrrolo[2,3-*c*]pyridine (**2b**)

Conditions: reflux for 1 h. Yellow solid; yield: 65%; mp: 216–217 °C; IR 3396 (NH) cm^−1^; ^1^H NMR (200 MHz, DMSO-*d_6_*) δ: 3.87 (s, 3H, CH_3_), 3.91 (s, 3H, OCH_3_), 6.95 (dd, 1H, *J* = 2.4, 8.9 Hz, H-6′), 7.49 (d, 1H, *J* = 8.9 Hz, H-7′), 7.79 (s, 1H, H-2′), 7.90 (d, 1H,* J* = 2.4 Hz, H-4′), 8.05 (d, 1H, *J* = 5.5 Hz, H-4″), 8.17 (s, 1H, H-5), 8.32 (d, 1H, *J* = 2.7 Hz, H-2″), 8.37 (d, 1H, *J* = 5.5 Hz, H-5″), 12.46 (bs, 1H, NH); ^13^C NMR (50 MHz, DMSO-*d_6_*) δ: 33.0 (q), 55.1 (q), 96.3 (s), 101.8 (d), 107.7 (d), 108.6 (s), 110.1 (s), 111.6 (d), 112.6 (d), 115.4 (d), 124.8 (s), 129.9 (d), 131.0 (d), 132.1 (s), 132.8 (s), 134.2 (s), 136.8 (d), 146.6 (s), 155.1 (s), 162.8 (s). Anal. Calcd for: C_20_H_15_ClN_4_OS: C, 60.83; H, 3.83; N, 14.19. Found: C, 60.62; H, 3.99; N, 13.99.

##### 3-[2-(5-Bromo-1-methyl-1*H*-indol-3-yl)-1,3-thiazol-4-yl]-7-chloro-1*H*-pyrrolo[2,3-*c*]pyridine (**2c**)

Conditions: reflux for 1 h. Yellow solid; yield: 93%; mp: 374–375 °C; IR 3410 (NH) cm^−1^; ^1^H NMR (200 MHz, DMSO-*d_6_*) δ: 3.89 (s, 3H, CH_3_), 7.42 (dd, 1H, *J* = 1.8, 8.7 Hz, H-6′), 7.57 (d, 1H,* J* = 8.7 Hz, H-7′), 7.84 (s, 1H, H-2′), 8.09 (d, 1H,* J* = 5.6 Hz, H-4″), 8.28 (s, 1H, H-5), 8.30 (d, 1H, *J* = 5.6 Hz, H-5″), 8.34 (d, 1H, *J* = 2.8 Hz, H-2″), 8.49 (d, 1H, *J* = 1.8 Hz, H-4′), 12.56 (bs, 1H, NH); ^13^C NMR (50 MHz, DMSO-*d_6_*) δ: 33.1 (q), 108.4 (d), 108.9 (s), 112.5 (s), 112.8 (d), 113.8 (s), 115.2 (d), 122.7 (d), 124.9 (d), 126.1 (s), 129.8 (s), 130.1 (d), 131.7 (s), 131.9 (d), 133.5 (s), 135.8 (s), 136.7 (d), 148.5 (s), 161.5 (s). Anal. Calcd for: C_19_H_12_BrClN_4_S: C, 51.43; H, 2.73; N, 12.63. Found: C, 51.17; H, 2.62; N, 12.88.

##### 7-Chloro-3-[2-(5-fluoro-1-methyl-1*H*-indol-3-yl)-1,3-thiazol-4-yl]-1*H*-pyrrolo[2,3-*c*]pyridine (**2d**)

Conditions: reflux for 1 h. Yellow solid; yield: 97%; mp: 337–338 °C; IR 3509 (NH) cm^−1^; ^1^H NMR (200 MHz, DMSO-*d_6_*) δ: 3.91 (s, 3H, CH_3_), 7.18 (td, 1H, *J* = 2.5, 9.2, 11.7 Hz, H-6′), 7.61 (dd, 1H, *J* = 4.5, 9.2 Hz, H-7′), 7.82 (s, 1H, H-2′), 8.02 (dd, 1H, *J* = 2.5, 11.7 Hz, H-4′), 8.09 (d, 1H, *J* = 5.6 Hz, H-4″), 8.25 (d, 1H, *J* = 5.6 Hz, H-5″), 8.30 (s, 1H, H-5), 8.34 (d, 1H, *J* = 2.8 Hz, H-2″), 12.50 (bs, 1H, NH). Anal. Calcd for: C_19_H_12_ClFN_4_S: C, 59.61; H, 3.16; N, 14.63. Found: C, 59.34; H, 3.21; N, 14.47.

##### 7-Chloro-1-methyl-3-[2-(1-methyl-1*H*-indol-3-yl)-1,3-thiazol-4-yl]-1*H*-pyrrolo[2,3-*c*]pyridine (**2e**)

Conditions: reflux for 1 h. Yellow solid; yield: 96%; mp: 265–266 °C; ^1^H NMR (200 MHz, DMSO-*d_6_*) δ: 3.91 (s, 3H, CH_3_), 4.24 (s, 3H, CH_3_), 7.27–7.36 (m, 2H, H-5′, H-6′), 7.57–7.61 (m, 1H, H-7′), 7.75 (s, 1H, H-2′), 8.06 (d, 1H, *J* = 5.5 Hz, H-4″), 8.22 (s, 1H, H-2″), 8.25 (d, 1H, *J* = 5.5 Hz, H-5″), 8.33 (s, 1H, H-5), 8.35–8.38 (m, 1H, H-4′). Anal. Calcd for: C_20_H_15_ClN_4_S: C, 63.40; H, 3.99; N, 14.79. Found: C, 63.14; H, 3.75; N, 14.59.

##### 7-Chloro-3-[2-(5-methoxy-1-methyl-1*H*-indol-3-yl)-1,3-thiazol-4-yl]-1-methyl-1*H*-pyrrolo[2,3-*c*]pyridine (**2f**)

Conditions: reflux for 1 h. Yellow solid; yield: 79%; mp: 231–232 °C; ^1^H NMR (200 MHz, DMSO-*d_6_*) δ: 3.87 (s, 3H, CH_3_), 3.90 (s, 3H, OCH_3_), 4.22 (s, 3H, CH_3_), 6.94 (d, 1H, *J* = 8.8 Hz, H-6′), 7.48 (d, 1H, *J* = 8.8 Hz, H-7′), 7.69 (s, 1H, H-2′), 7.87–7.91 (m, 1H, H-4′), 8.02 (d, 1H, *J* = 5.4 Hz, H-4″), 8.15 (s, 1H, H-2″), 8.28 (s, 1H, H-5), 8.35 (d, 1H, *J* = 5.4 Hz, H-5″). Anal. Calcd for: C_21_H_17_ClN_4_OS: C, 61.68; H, 4.19; N, 13.70. Found: C, 61.42; H, 4.08; N, 13.47.

##### 3-[2-(5-Bromo-1-methyl-1*H*-indol-3-yl)-1,3-thiazol-4-yl]-7-chloro-1-methyl-1*H*-pyrrolo[2,3-*c*]pyridine (**2g**)

Conditions: reflux for 2 h. Yellow solid; yield: 98%; mp: 249–250 °C; ^1^H NMR (200 MHz, DMSO-*d_6_*) δ: 3.90 (s, 3H, CH_3_), 4.23 (s, 3H, CH_3_), 7.44 (dd, 1H,* J* = 1.9, 8.7 Hz, H-6′), 7.58 (d, 1H, *J* = 8.7 Hz, H-7′), 7.74 (s, 1H, H-2′), 8.04 (d, 1H,* J* = 5.5 Hz, H-4″), 8.24–8.28 (m, 3H, H-2″,H-5, H-5″), 8.48 (d, 1H, *J* = 1.9 Hz, H-4′); ^13^C NMR (50 MHz, DMSO-*d_6_*) δ: 33.1 (q), 36.0 (q), 108.4 (d), 109.0 (s), 110.4 (s), 112.8 (d), 113.8 (s), 115.3 (d), 122.8 (d), 124.9 (d), 126.0 (s), 128.8 (s), 131.9 (d), 133.0 (s), 133.3 (s), 135.3 (d), 135.8 (s), 137.0 (d), 148.1 (s), 161.5 (s). Anal. Calcd for: C_20_H_14_BrClN_4_S: C, 52.47; H, 3.08; N, 12.24. Found: C, 52.23; H, 2.94; N, 12.11.

##### 7-Chloro-3-[2-(5-fluoro-1-methyl-1*H*-indol-3-yl)-1,3-thiazol-4-yl]-1-methyl-1*H*-pyrrolo[2,3-*c*]pyridine (**2h**)

Conditions: reflux for 1 h. Yellow solid; yield: 95%; mp: 284–285 °C; ^1^H NMR (200 MHz, DMSO-*d_6_*) δ: 3.91 (s, 3H, CH_3_), 4.24 (s, 3H, CH_3_), 7.18 (td, 1H, *J* = 2.5, 9.3, 11.7 Hz, H-6′), 7.61 (dd, 1H,* J* = 4.4, 9.3 Hz, H-7′), 7.75 (s, 1H, H-2′), 8.02–8.07 (m, 2H, H-4′, H-4″), 8.22 (d, 1H, *J* = 5.6 Hz, H-5″), 8.28 (s, 1H, H-2″), 8.35 (s, 1H, H-5). Anal. Calcd for: C_20_H_14_ClFN_4_S: C, 60.53; H, 3.56; N, 14.12. Found: C, 60.29; H, 3.30; N, 14.27.

##### 7-Chloro-3-[2-(5-fluoro-1*H*-indol-3-yl)-1,3-thiazol-4-yl]-1-methyl-1*H*-pyrrolo[2,3-*c*]pyridine (**2i**)

Conditions: reflux for 3 h. Yellow solid; yield: 82%; mp: 284–285 °C; IR 3399 (NH) cm^−1^; ^1^H NMR (200 MHz, DMSO-*d_6_*) δ: 4.25 (s, 3H, CH_3_), 7.11 (td, 1H, *J* = 2.5, 9.2, 11.7 Hz, H-6′), 7.53 (dd, 1H,* J* = 4.6, 9.2 Hz, H-7′), 7.75 (s, 1H, H-2″), 8.02 (dd, 1H, *J* = 2.5, 11.7 Hz, H-4′), 8.06 (d, 1H, *J* = 5.5 Hz, H-4″), 8.22 (d, 1H, *J* = 5.5 Hz, H-5″), 8.25 (d, 1H, *J* = 2.9 Hz, H-2′), 8.35 (s, 1H, H-5), 11.93 (bs, 1H, NH); ^13^C NMR (50 MHz, DMSO-*d_6_*) δ: 36.6 (q), 105.2 (d, *J_C4′-F_* = 24.3 Hz), 108.2 (d), 110.5 (s), 110.7 (d, *J_C6′-F_* = 26.0 Hz), 113.4 (d, *J_C7′-F_* = 9.6 Hz), 115.1 (s), 115.2 (d), 124.5 (s, *J_C3a–f_* = 10.8 Hz), 128.7 (d), 128.9 (s), 132.9 (s), 133.2 (s), 133.4 (s), 135.8 (d), 137.0 (d), 137.5 (s), 148.1 (s), 159.7 (s, *J_C5′-F_* = 254 Hz). Anal. Calcd for: C_19_H_12_ClFN_4_S: C, 59.61; H, 3.16; N, 14.63. Found: C, 59.37; H, 2.90; N, 14.83.

##### 7-Chloro-3-[2-(5-fluoro-1*H*-indol-3-yl)-1,3-thiazol-4-yl]-1*H*-pyrrolo[2,3-*c*]pyridine (**2j**)

Conditions: reflux for 2 h. Yellow solid; yield: 66%; mp: 281–282 °C; IR 3418, 3557 (NH) cm^−1^; ^1^H NMR (200 MHz, DMSO-*d_6_*) δ: 7.12 (td, 1H, *J* = 2.5, 9.2, 11.7 Hz, H-6′), 7.53 (dd, 1H, *J* = 4.6, 9.2 Hz, H-7′), 7.83 (s, 1H, H-2′), 8.00 (dd, 1H, *J* = 2.5, 11.7 Hz, H-4′), 8.08 (d, 1H, *J* = 5.6 Hz, H-4″), 8.24–8.29 (m, 2H, H-5, H-5″), 8.32 (d, 1H, *J* = 2.8 Hz, H-2″), 11.95 (bs, 1H, NH), 12.45 (bs, 1H, NH). Anal. Calcd for: C_18_H_10_ClFN_4_S: C, 58.62; H, 2.73; N, 15.19. Found: C, 58.51; H, 2.47; N, 14.94.

##### *Tert*-Butyl 3-[4-(7-Chloro-1*H*-pyrrolo[2,3-*c*]pyridin-3-yl)-1,3-thiazol-2-yl]-1*H*-indole-1-carboxylate (**3a**)

Conditions: reflux for 2 h. Yellow solid; yield: 84%, mp: 225–226 °C; IR 3417 (NH), 1736 (CO) cm^−1^; ^1^H NMR (200 MHz, DMSO-*d_6_*) δ: 1.69 (s, 9H, 3xCH_3_), 7.44–7.53 (m, 2H, H-5′, H-6′), 8.02 (s, 1H, H-2′), 8.09 (d, 1H, *J* = 5.5 Hz, H-4″), 8.16–8.21 (m, 1H, H-7′), 8.23 (d, 1H, *J* = 5.5 Hz, H-5″), 8.35–8.36 (m, 2H, H-2″, H-5), 8.47–8.51 (m, 1H, H-4′), 12.48 (bs, 1H, NH); ^13^C NMR (50 MHz, DMSO-*d_6_*) δ: 27.6 (3xq), 84.9 (s), 110.4 (d), 112.2 (s), 114.9 (s), 115.0 (d), 121.3 (d), 123.8 (d), 123.9 (d), 125.4 (d), 125.6 (d), 126.6 (s), 129.8 (s), 129.9 (d), 131.4 (s), 133.8 (s), 135.0 (s), 137.5 (d), 148.6 (s), 149.3 (s), 159.9 (s). Anal. Calcd for: C_23_H_19_ClN_4_O_2_S: C, 61.26; H, 4.25; N, 12.42. Found: C, 61.02; H, 4.06; N, 12.13.

##### *Tert*-Butyl 3-[4-(7-chloro-1*H*-pyrrolo[2,3-*c*]pyridin-3-yl)-1,3-thiazol-2-yl]-5-methoxy-1*H*-indole-1-carboxylate (**3b**)

Conditions: reflux for 1 h. Yellow solid; yield: 77%; mp: 312–313 °C; IR 3418 (NH), 1650 (CO) cm^−1^; ^1^H NMR (200 MHz, DMSO-*d_6_*) δ: 1.68 (s, 9H, 3xCH_3_), 3.92 (s, 3H, OCH_3_), 7.09 (dd, 1H, *J* = 2.4, 9.1 Hz, H-6′), 7.99–8.07 (m, 4H, ArH), 8.30–8.35 (m, 3H, ArH), 12.47 (bs, 1H, NH); ^13^C NMR (50 MHz, DMSO-*d_6_*) δ: 27.6 (3xq), 55.1 (q), 84.7 (s), 103.2 (d), 110.2 (d), 112.2 (s), 114.5 (d), 114.8 (s), 115.2 (d), 115.8 (d), 125.9 (d), 127.5 (s), 129.5 (s), 130.0 (d), 131.6 (s), 133.7 (s), 136.9 (d), 137.2 (s), 148.5 (s), 149.2 (s), 156.1 (s), 160.2 (s). Anal. Calcd for: C_24_H_21_ClN_4_O_3_S: C, 59.93; H, 4.40; N, 11.65. Found: C, 59.66; H, 4.61; N, 11.50.

##### *Tert*-Butyl 5-bromo-3-[4-(7-chloro-1*H*-pyrrolo[2,3-*c*]pyridin-3-yl)-1,3-thiazol-2-yl]-1*H*-indole-1-carboxylate (**3c**)

Conditions: reflux for 3 h. Yellow solid; yield: 77%, mp: 241–242 °C; IR 3559 (NH), 1748 (CO) cm^−1^; ^1^H NMR (200 MHz, DMSO-*d_6_*) δ: 1.67 (s, 9H, 3xCH_3_), 7.60 (d, 1H, *J* = 8.6 Hz, H-6′), 7.98 (s, 1H, H-2′), 8.04–8.07 (m, 2H, H-2″, H-7′ ), 8.19 (d, 1H, *J* = 5.3 Hz, H-4″), 8.29–8.33 (m, 2H, H-4′, H-5″), 8.65 (s, 1H, H-5), 12.45 (bs, 1H, NH); ^13^C NMR (50 MHz, DMSO-*d_6_*) δ: 27.6 (3xq), 85.3 (s), 110.5 (d), 112.0 (s), 114.1 (s), 114.9 (d), 116.8 (d), 123.8 (d), 126.8 (d), 127.9 (d), 128.4 (s), 129.5 (d), 129.8 (s), 129.9 (s), 131.4 (s), 133.8 (s), 134.0 (s), 137.4 (d), 148.3 (s), 149.3 (s), 159.4 (s). Anal. Calcd for: C_23_H_18_BrClN_4_O_2_S: C, 52.14; H, 3.42; N, 10.57. Found: C, 52.03; H, 3.55; N, 10.32.

##### *Tert*-Butyl 3-[4-(7-chloro-1-methyl-1*H*-pyrrolo[2,3-*c*]pyridin-3-yl)-1,3-thiazol-2-yl]-1*H*-indole-1-carboxylate (**3d**)

Conditions: reflux for 2 h. Yellow solid; yield: 91%; mp: 279–280 °C; IR 1736 (CO) cm^−1^; ^1^H NMR (200 MHz, DMSO-*d_6_*) δ: 1.69 (s, 9H, 3xCH_3_), 4.24 (s, 3H, CH_3_), 7.44–7.54 (m, 2H, H-5′, H-6′), 7.96 (s, 1H, H-2′), 8.07 (d, 1H, *J* = 5.5 Hz, H-4″), 8.16–8.20 (m, 1H, H-7′), 8.20 (d, 1H, *J* = 5.5 Hz, H-5″), 8.34 (s, 1H, H-2″), 8.37 (s, 1H, H-5), 8.48–8.52 (m, 1H, H-4′); ^13^C NMR (50 MHz, DMSO-*d_6_*) δ: 27.6 (3xq), 36.6 (q), 84.9 (s), 110.2 (s), 110.4 (d), 114.9 (s), 115.0 (d), 115.1 (d), 121.4 (d), 123.8 (d), 125.5 (d), 125.6 (d), 126.6 (s), 128.9 (s), 132.8 (s), 133.3 (s), 135.0 (s), 135.7 (d), 137.4 (d), 148.6 (s), 149.6 (s), 159.9 (s). Anal. Calcd for: C_24_H_21_ClN_4_O_2_S: C, 62.00; H, 4.55; N, 12.05. Found: C, 61.73; H, 4.41; N, 11.94.

##### *Tert*-Butyl 3-[4-(7-chloro-1-methyl-1*H*-pyrrolo[2,3-*c*]pyridin-3-yl)-1,3-thiazol-2-yl]-5-methoxy-1*H*-indozle-1-carboxylate (**3e**)

Conditions: reflux for 1 h. Yellow solid; yield: 92%; mp: 212–213 °C; IR 1723 (CO) cm^−1^; ^1^H NMR (200 MHz, DMSO-*d_6_*) δ: 1.68 (s, 9H, 3xCH_3_), 3.91 (s, 3H, OCH_3_), 4.21 (s, 3H, CH_3_), 7.08 (d, 1H, *J* = 9.0 Hz, H-6′), 7.89–8.06 (m, 4H, ArH), 8.27–8.29 (m, 3H, ArH). Anal. Calcd for: C_25_H_23_ClN_4_O_3_S: C, 60.66; H, 4.68; N, 11.32. Found: C, 60.45; H, 4.89; N, 11.56.

##### *Tert*-Butyl 5-bromo-3-[4-(7-chloro-1-methyl-1*H*-pyrrolo[2,3-*c*]pyridin-3-yl)-1,3-thiazol-2-yl]-1*H*-indole-1-carboxylate (**3f**)

Conditions: reflux for 3 h. Yellow solid; yield: 91%; mp: 257 °C; IR 1727 (CO) cm^−1^; ^1^H NMR (200 MHz, DMSO-*d_6_*) δ: 1.69 (s, 9H, 3xCH_3_), 4.22 (s, 3H, CH_3_), 7.63 (dd, 1H, *J* = 1.9, 8.9 Hz, H-6′), 7.91 (s, 1H, H-2′), 8.03 (d, 1H, *J* = 5.5 Hz, H-4″), 8.09 (d, 1H, *J* = 8.9 Hz, H-7′), 8.18 (d, 1H, *J* = 5.5 Hz, H-5″), 8.27 (s, 1H, H-2″), 8.35 (s, 1H, H-5), 8.61 (d, 1H, *J* = 1.9 Hz, H-4′). Anal. Calcd for: C_24_H_20_BrClN_4_O_2_S: C, 53.00; H, 3.71; N, 10.30. Found: C, 53.27; H, 3.97; N, 10.16.

#### 3.1.11. General Procedure for the Synthesis of Indolyl-thiazolyl-pyrrolo[2,3-*c*]pyridines (**4a–f**)

To a suspension of appropriate *N*-*tert*-butylcarboxylate derivatives **3a–f** (0.78 mmol) in DCM (10 mL) TFA (1.1 mL) was added. The reaction was heated under reflux for 24 h. The mixture was neutralized with saturated aqueous NaHCO_3_ solution. The solvent was dried (Na_2_SO_4_), evaporated under reduced pressure and the residue recrystallized from EtOH to afford indolyl-thiazolyl-pyrrolo[2,3-*c*]pyridines **4a–f**.

##### 7-Chloro-3-[2-(1*H*-indol-3-yl)-1,3-thiazol-4-yl]-1*H*-pyrrolo[2,3-*c*]pyridine (**4a**)

Green solid; yield: 93%; mp: 271–272 °C; IR 3556, 3394 (NH) cm^−1^; ^1^H NMR (200 MHz, DMSO-*d_6_*) δ: 7.21–7.31 (m, 2H, H-5′, H-6′), 7.48–7.55 (m, 1H, H-7′), 7.81 (s, 1H, H-2′), 8.08 (d, 1H, *J* = 5.5 Hz, H-4″), 8.20 (d, 1H, *J* = 2.8 Hz, H-2″), 8.27–8.35 (m, 3H, H-5, H-4′, H-5″), 11.82 (bs, 1H, NH), 12.41 (bs, 1H, NH); ^13^C NMR (50 MHz, DMSO-*d_6_*) δ: 108.0 (d), 110.4 (s), 112.2 (d), 112.5 (s), 115.3 (d), 120.2 (d), 120.8 (d), 122.4 (d), 124.2 (s), 126.8 (d), 129.4 (d), 129.8 (s), 131.4 (s), 133.9 (s), 136.6 (s), 137.5 (d), 148.6 (s), 162.5 (s). Anal. Calcd for: C_18_H_11_ClN_4_S: C, 61.62; H, 3.16; N, 15.97. Found: C, 61.47; H, 3.40; N, 15.82.

##### **7**-Chloro-3-[2-(5-methoxy-1*H*-indol-3-yl)-1,3-thiazol-4-yl]-1*H*-pyrrolo[2,3-*c*]pyridine (**4b**)

Yellow solid; yield: 62%; mp: 223–224 °C; IR 3555, 3379 (NH) cm^−1^; ^1^H NMR (200 MHz, DMSO-*d_6_*) δ: 3.90 (s, 3H, OCH_3_), 6.89 (dd, 1H,* J* = 2.5, 8.8 Hz, H-6′), 7.42 (d, 1H, *J* = 8.8 Hz, H-7′), 7.80 (s, 1H, H-5), 7.89 (d, 1H, *J* = 2.5 Hz, H-4′), 8.07 (d, 1H, *J* = 5.6 Hz, H-4″), 8.15 (d, 1H, *J* = 2.9 Hz, H-2′), 8.35 (d, 1H, *J* = 2.8 Hz, H-2″), 8.39 (d, 1H, *J* = 5.6 Hz, H-5″), 11.72 (bs, 1H, NH), 12.51 (bs, 1H, NH); ^13^C NMR (50 MHz, DMSO-*d_6_*) δ: 55.1 (q), 101.6 (d), 107.7 (d), 110.1 (s), 112.4 (s), 112.7 (d), 113.0 (d), 115.4 (d), 124.7 (s), 127.2 (d), 129.6 (d), 129.8 (s), 131.5 (s), 131.7 (s), 133.7 (s), 137.0 (d), 148.3 (s), 154.7 (s), 162.9 (s). Anal. Calcd for: C_19_H_13_ClN_4_OS: C, 59.92; H, 3.44; N, 14.71. Found: C, 59.68; H, 3.33; N, 14.56.

##### 3-[2-(5-Bromo-1*H*-indol-3-yl)-1,3-thiazol-4-yl]-7-chloro-1*H*-pyrrolo[2,3-*c*]pyridine (**4c**)

Yellow solid; yield: 98%; mp: 265–266 °C; IR 3395, 3124 (NH) cm^−1^; ^1^H NMR (200 MHz, DMSO-*d_6_*) δ: 7.37 (dd, 1H, *J* = 1.9, 8.6 Hz, H-6′), 7.51 (d, 1H, *J* = 8.6 Hz, H-7′), 7.84 (bs, 1H, H-2″), 8.08 (d, 1H, *J* = 5.6 Hz, H-4″), 8.24–8.28 (m, 2H, H-5, H-5″), 8.32 (d, 1H, *J* = 2.9 Hz, H-2′), 8.53 (d, 1H, *J* = 1.9 Hz, H-4′), 12.02 (bs, 1H, NH), 12.48 (bs, 1H, NH); ^13^C NMR (50 MHz, DMSO-*d_6_*) δ: 108.3 (d), 110.0 (s), 112.5 (s), 113.4 (s), 114.3 (d), 115.2 (d), 112.6 (d), 124.9 (d), 126.0 (s), 128.2 (d), 129.6 (d), 129.8 (s), 131.6 (s), 133.8 (s), 135.3 (s), 137.1 (d), 148.6 (s), 161.9 (s). Anal. Calcd for: C_18_H_10_BrClN_4_S: C, 50.31; H, 2.35; N, 13.04. Found: C, 50.55; H, 2.09; N, 12.88.

##### 7-Chloro-3-[2-(1*H*-indol-3-yl)-1,3-thiazol-4-yl]-1-methyl-1*H*-pyrrolo[2,3-*c*]pyridine (**4d**)

Green solid; yield: 75%; mp: 210–211 °C; IR 3397 (NH) cm^−1^; ^1^H NMR (200 MHz, DMSO-*d_6_*) δ: 4.25 (s, 3H, CH_3_), 7.22–7.30 (m, 2H, H-6′, H-5′), 7.48–7.54 (m, 1H, H-7′), 7.75 (s, 1H, H-2″), 8.06 (d, 1H, *J* = 5.5 Hz, H-4″), 8.18 (d, 1H, *J* = 2.8 Hz, H-2′), 8.26 (d, 1H,* J* = 5.5 Hz, H-5″), 8.33 (s, 1H, H-5), 8.36–8.39 (m, 1H, H-4′), 11.82 (bs, 1H, NH); ^13^C NMR (50 MHz, DMSO-*d_6_*) δ: 36.6 (q), 108.0 (d), 110.4 (s), 110.6 (s), 112.2 (d), 115.3 (d), 120.3 (d), 120.8 (d), 122.4 (d), 124.2 (s), 126.7 (d), 128.9 (s), 132.9 (s), 133.4 (s), 136.6 (s), 135.2 (d), 137.6 (d), 148.0 (s), 162.5 (s). Anal. Calcd for: C_19_H_13_ClN_4_S: C, 62.55; H, 3.59; N, 15.36. Found: C, 62.31; H, 3.45; N, 15.21.

##### 7-Chloro-3-[2-(5-methoxy-1*H*-indol-3-yl)-1,3-thiazol-4-yl]-1-methyl-1*H*-pyrrolo[2,3-*c*]pyridine (**4e**)

Orange solid; yield: 73%; mp: 184–185 °C; IR 3383 (NH) cm^−1^; ; ^1^H NMR (200 MHz, DMSO-*d_6_*) δ: 3.89 (s, 3H, OCH_3_), 4.22 (s, 3H, CH_3_), 6.88 (dd, 1H, *J* = 2.5, 8.8 Hz, H-6′), 7.41 (d, 1H, *J* = 8.8 Hz, H-7′), 7.69 (s, 1H, H-2″), 7.87 (d, 1H, *J* = 2.5 Hz, H-4′), 8.02 (d, 1H, *J* = 5.5 Hz, H-4″), 8.11 (d, 1H,* J* = 2.9 Hz, H-2′), 8.28 (s, 1H, H-5), 8.36 (d, 1H, *J* = 5.5 Hz, H-5″), 11.67 (bs, 1H, NH); ^13^C NMR (50 MHz, DMSO-*d_6_*) δ: 36.6 (q), 55.1 (q), 101.7 (d), 107.6 (d), 110.2 (s), 110.5 (s), 112.6 (d), 113.0 (d), 115.5 (d), 124.7 (s), 127.1 (d), 128.9 (s), 131.5 (s), 133.1 (s), 133.4 (s), 134.9 (d), 137.3 (d), 148.0 (s), 154.6 (s), 162.8 (s). Anal. Calcd for: C_20_H_15_ClN_4_OS: C, 60.83; H, 3.83; N, 14.19. Found: C, 60.59; H, 3.72; N, 13.94.

##### 3-[2-(5-Bromo-1*H*-indol-3-yl)-1,3-thiazol-4-yl]-7-chloro-1-methyl-1*H*-pyrrolo[2,3-*c*]pyridine (**4f**)

Green solid; yield: 99%; mp: 273–274 °C; IR 3378 (NH) cm^−1^; 1H NMR (200 MHz, DMSO-*d_6_*) δ: 4.24 (s, 3H, CH_3_), 7.37 (dd, 1H, *J* = 1.9, 8.6 Hz, H-6′), 7.50 (d, 1H, *J* = 8.6 Hz, H-7′), 7.75 (s, 1H, H-2″), 8.04 (d, 1H, *J* = 5.5 Hz, H-4″), 8.24–8.29 (m, 3H, H-2′, H-5, H-5″), 8.50 (d, 1H, *J* = 1.9 Hz, H-4′), 12.02 (bs, 1H, NH); ^13^C NMR (50 MHz, DMSO-*d_6_*) δ: 36.6 (q), 108.4 (d), 110.0 (s), 110.5 (s), 113.4 (s), 114.3 (d), 115.3 (d), 117.8 (s), 122.5 (d), 125.0 (d), 125.9 (s), 128.2 (d), 128.9 (s), 133.1 (s), 133.3 (s), 135.2 (d), 137.2 (d), 148.1 (s), 162.0 (s). Anal. Calcd for: C_19_H_12_BrClN_4_S: C, 51.43; H, 2.73; N, 12.63. Found: C, 51.19; H, 2.60; N, 12.47.

### 3.2. Biology

All reagents and chemicals were from Sigma Chemical Co (St. Louis, MO, USA), unless indicated.

#### 3.2.1. Viability Assay *in Vitro*

Nortopsentin analogues, prepared as described above, were dissolved in dimethyl sulfoxide (DMSO) and then diluted in culture medium to have a DMSO concentration not exceeding 0.1%. HCT-116 and Caco-2 cell lines were purchased from American Type Culture Collection, Rockville, MD, USA and DMEM supplemented with 10% fetal, 10% fetal bovine serum (FBS), penicillin (100 U/mL), streptomycin (100 μg/mL) and gentamicin (5 μg/mL). Cells were maintained in log phase by seeding twice a week at a density of 3 × 10^8^ cells/L in humidified 5% CO2 atmosphere, at 37 °C. In all experiments, HCT-116 cells were made quiescent through overnight incubation before treatment with tested compounds or vehicle alone (control cells), while Caco-2 cells were treated 15 days after confluence, at which time the cells are differentiated in normal intestinal-like cells [53]. No differences were found between cells treated with DMSO 0.1% and untreated cells in terms of cell number and viability.

Cytotoxic activity of the nortopsentin derivatives **1k** and **4c** was determined by the colorimetric assay based on the reduction of 3-(4,5-dimethyl-2-thiazolyl)bromide-2,5-diphenyl-2*H*-tetrazolium (MTT) to purple formazan by mitochondrial dehydrogenases. Briefly, HCT-116 and Caco-2 lines cells were seeded at 2 × 10^4^ cells/well in 96-well plates containing 200 μL RPMI. When appropriated, monolayer cultures were treated for 24 h with various concentrations (5–100 μM) of the drugs. Then cells were washed with fresh medium and 50 μL FBS-free medium containing 5 mg/mL MTT added. Cells were incubated 2 h at 37 °C, then medium was discarded by centrifugation, formazan blue formed in the cells dissolved in DMSO, and absorbance measured at 570 nm in a microplate reader (Bio-RAD, Hercules, CA, USA). Formazan of control cells was taken as 100% viability. GI_50_ was calculated by the curve of percent viability* vs.* concentration. Each experiment was repeated at least three times in triplicate.

#### 3.2.2. Cell Cycle Analysis

Cell cycle stage was analyzed by flow cytometry. HCT-116 cells (5.0 × 10^4^ cells/cm^2^) were seeded in triplicate in 24-wells culture plates. After an overnight incubation, the cells were washed with fresh medium and incubated with compounds **1k** and **4c** in RPMI for 24 h. Then cells were harvested by trypsinization. Aliquots of 1 × 10^6^ cells were washed with PBS and incubated in the dark in a PBS solution containing 20 μg/mL propidium iodide (PI) and 200 μg/mL RNase, for 30 min, at room temperature. Then samples of at least 1.0 × 10^4^ cells were subjected to fluorescence-activated cell sorting (FACS) analysis by Epics XL™ flow cytometer using Expo32 software (Beckman Coulter, Fullerton, CA, USA).

#### 3.2.3. Measurement of Phosphatidylserine (PS) Exposure

The apoptosis-induced PS externalization to the cell surface was measured by flow cytometry by double staining with Annexin V-Fluorescein isothiocyanate (Annexin V-FITC)/propidium iodide (PI). Annexin V binding to phosphatidylserine is used to identify the earliest stage of apoptosis. PI, which does not enter cells with intact membranes, is used to distinguish between early apoptotic cells (Annexin V-FITC positive and PI negative), late apoptotic cells (Annexin V-FITC/PI-double positive) or necrotic cells (annexin V-FITC negative and PI positive). After 24 h treatment, HCT-116 cells were harvested by trypsinization and adjusted at 1.0 × 10^6^ cells/mL with combining buffer according to the manufacturer instructions (eBioscience, San Diego, CA, USA). One hundred μL of cell suspensions were added to a new tube, and incubated with Annexin V-FITC and PI solution at room temperature in the dark for 15 min. Then samples of at least 1.0 × 10^4^ cells were subjected to FACS analysis using appropriate 2-bidimensional gating method.

#### 3.2.4. Measurement of Mitochondrial Transmembrane Potential

Changes of mitochondrial transmembrane potential (Δψm) were assessed by flow cytofluorometry, using the cationic lipophilic dye 3,3′-dihexyloxacarbocyanine iodide(3) (DiOC6) (Molecular Probes, Inc., Life Technologies Italia, Monza, Italy) which accumulates in the mitochondrial matrix. Changes in mitochondrial membrane potential are indicated by a reduction in the DiOC6-induced fluorescence intensity. After 24 h treatment, HCT116 cells were incubated with DiOC6 at a 40 nmol/L final concentration, for 15 min at 37 °C. After centrifugation, cells were washed with PBS and suspended in 500 μL PBS. Fluorescent intensities were analysed in at least 1 × 10^4^ cells for each sample.

#### 3.2.5. Morphology

To analyse cell morphology, cells were fixed with methanol for 10 min and then stained with Giemsa (10% in PBS) for 15 min followed by washing with distilled water. Cell images were captured using Zeiss, AxioCam, AxioSkop microscope, (West Germany) with 40× lenses.

#### 3.2.6. Quantification of Acidic Vesicular Organelles (AVO) by Acridine Orange (AO) Staining

AO is a fluorescent molecule used either to identify apoptotic cell death or autophagy. It can interact with DNA emitting green fluorescence or accumulate in acidic organelles where it is protonated forming aggregates that emit bright red fluorescence [52]. Briefly, cells were stained with a AO solution (5 μg/mL) for 15 min. Then they were washed, re-suspended in PBS and subjected to FACS analysis. The green (510–530 nm, FL-1) and red (650 nm, FL-3) fluorescence of AO with blue (488 nm) excitation was determined over 10,000 events.

#### 3.2.7. Statistics

Results are given as means and their standard deviations. Three independent observations were carried out for each experiment, replicated three times. Statistical comparisons were made using a one-way ANOVA, followed by Bonferroni’s test. *p* < 0.05 was considered statistically significant.

## 4. Conclusions

Two new series of nortopsentin analogues were conveniently prepared. In these series the imidazole ring of the natural product was replaced by a thiazole, and, in one case, both indole units were substituted by 7-azaindole moieties; in the other series, one indole moiety was replaced by a 6-azaindole system.

The newly synthesized nortopsentin analogues **1k** and **4c** showed cytotoxicity towards a broad spectrum of human cancer cell lines included in the NCI panel. Importantly, the compounds did not appreciably impair vitality of intestinal normal like cells. Investigating mechanisms underlying the antiproliferative effect in HCT-116 colon cancer cells, we showed that **1k** caused a dose-dependent increase of the apoptotic cell population, activating the mitochondria-mediated pathway and inducing cell cycle arrest at the G2/M phase. Comparably, previous work showed that other similar analogues caused apoptosis and arrest of cell cycle with inhibition of cyclin-dependent kinase 1 activity kinase [42,43,44]. On the other hand **4c** at a concentration lower than its GI_50_, induced antiproliferative effects with massive accumulation of autophagic vacuoles without apparent signs of apoptosis. The observed arrest of cell cycle at G1 phase, supports the autophagic fate of the cells [54]. Evolution of the cells toward apoptotic death following treatment with higher drug concentrations, suggests that **4c** may orchestrate a potential axis of autophagy and apoptosis in HCT-116 cells which can facilitate cellular destruction. The autophagy-signaling cascade induced by **4c** in HCT-116 cells is currently under investigation. Modulating authophagy appears of great interest in cancer. Indeed, current evidence suggests that authophagic cell death can be induced as an alternative to apoptosis with therapeutic purpose in cancer cells that are resistant to apoptosis. In this context, **4c** may candidate as a lead compound for nortopsentin derivatives with autophagic activity.
